# Hyperinvasive Meningococci Induce Intra-nuclear Cleavage of the NF-κB Protein p65/RelA by Meningococcal IgA Protease

**DOI:** 10.1371/journal.ppat.1005078

**Published:** 2015-08-04

**Authors:** Anissa Besbes, Salomé Le Goff, Ana Antunes, Aude Terrade, Eva Hong, Dario Giorgini, Muhamed-Kheir Taha, Ala-Eddine Deghmane

**Affiliations:** Institut Pasteur, Invasive Bacterial Infections Unit, Paris, France; University of Toronto, CANADA

## Abstract

Differential modulation of NF-κB during meningococcal infection is critical in innate immune response to meningococcal disease. Non-invasive isolates of *Neisseria meningitidis* provoke a sustained NF-κB activation in epithelial cells. However, the hyperinvasive isolates of the ST-11 clonal complex (ST-11) only induce an early NF-κB activation followed by a sustained activation of JNK and apoptosis. We show that this temporal activation of NF-κB was caused by specific cleavage at the C-terminal region of NF-κB p65/RelA component within the nucleus of infected cells. This cleavage was mediated by the secreted 150 kDa meningococcal ST-11 IgA protease carrying nuclear localisation signals (NLS) in its α-peptide moiety that allowed efficient intra-nuclear transport. In a collection of non-ST-11 healthy carriage isolates lacking NLS in the α-peptide, secreted IgA protease was devoid of intra-nuclear transport. This part of *iga* polymorphism allows non-invasive isolates lacking NLS, unlike hyperinvasive ST-11 isolates of *N*. *meningitides* habouring NLS in their α-peptide, to be carried asymptomatically in the human nasopharynx through selective eradication of their ability to induce apoptosis in infected epithelial cells.

## Author Summary

Strains of *Neisseria meningitidis* isolated from patients induce apoptotic cell death, whereas strains isolated from healthy carriage isolates do not. Part of the difference has been shown to arise from differential modulation of NF-κB during meningococcal infection. While non-invasive isolates of *Neisseria meningitidis* provoke a sustained NF-κB activation in epithelial cells, hyperinvasive isolates only induce an early NF-κB activation followed by a sustained activation of JNK and apoptosis. Here, we elucidate the mechanism conferring this differential modulation, specifically showing that ST-11 hyperinvasive isolates promote specific cleavage of NF-κB p65/RelA component in a manner dependent on the secreted IgA protease. This cleavage occurs within the nuclear compartment. Secreted IgA protease from non-invasive isolates was unable to reach the nuclear compartment of infected cells, resulting in a sustained activation of NF-κB activity and subsequent cytoprotective effect. Modulation of NF-κB-related signaling is likely a double-edged sword to decide the fate of meningococcal infection.

## Introduction

*Neisseria meningitidis* (Nm) is a leading cause of severe invasive infections mainly in children, leading to septicaemia and meningitis. The onset of these infections can be extremely rapid, leading to high morbidity and mortality despite appropriate antimicrobial chemotherapy and modern intensive care [1]. However, this pathogen is also frequently carried asymptomatically in about 10% of the general population as part of the commensal flora of the human nasopharynx [2]. A combination of host and bacterial factors may ultimately lead to meningococcal disease [3,4,5]. Indeed, only few meningococcal genetic lineages, referred to as hyperinvasive clonal complexes and rarely encountered in healthy carriers, are responsible for most cases of meningococcal disease [6,7]. Among these lineages, the clonal complex ST-11 (ST-11), that is most frequently of serogroup C, has been provoking outbreaks worldwide with high mortality rate [8,9] which has promoted the use of conjugate vaccine against serogroup C meningococci [10,11].

There is increasing evidence that invasive meningococcal infections lead to cytopathic effects that are consistent with the extensive cell injury and tissue damage [12,13,14,15]. We have previously shown a strong association between ST-11 isolates and apoptosis of infected epithelial cells [16,17] that required sustained activation of c-Jun N-terminal kinase (JNK) as a consequence of alteration of NF-κB activity [17,18]. In contrast carriage isolates promote a sustained cytoprotective NF-κB activity with only transient activation of JNK. The NF-κB consists of a heterodimeric complex composed of two subunits, commonly p50/NF-κB1, a DNA-binding subunit, and p65/RelA subunit which provides the transactivation activity of NF-κB. This heterodimeric complex is sequestered in the cytoplasm of resting cells and is rendered inactive through its association with the inhibitor of NF-κ (IκB) [19]. NF-κB-activating stimuli such as bacterial infection, proinflammatory cytokines or LPS, facilitate IκB kinase (IKK)-mediated IκB phosphorylation and subsequent degradation of IκB by the proteasome machinery [20], resulting in the release and subsequent nuclear translocation of the NF-κB complex for regulation of genes that are involved in the immunity process, adhesion molecules and cell survival [21]. We aimed in the present study to determine the mechanism leading to the differential impairment of NF-κB activity between invasive ST-11 isolates (referred to as ST-11 isolates) and carriage isolates (referred to as non-ST-11 isolates).

## Results

### Pathogenic meningococcal ST-11 isolates alter NF-κB activity by promoting nuclear cleavage of NF-κB p65/RelA subunit

**Fig 1:**
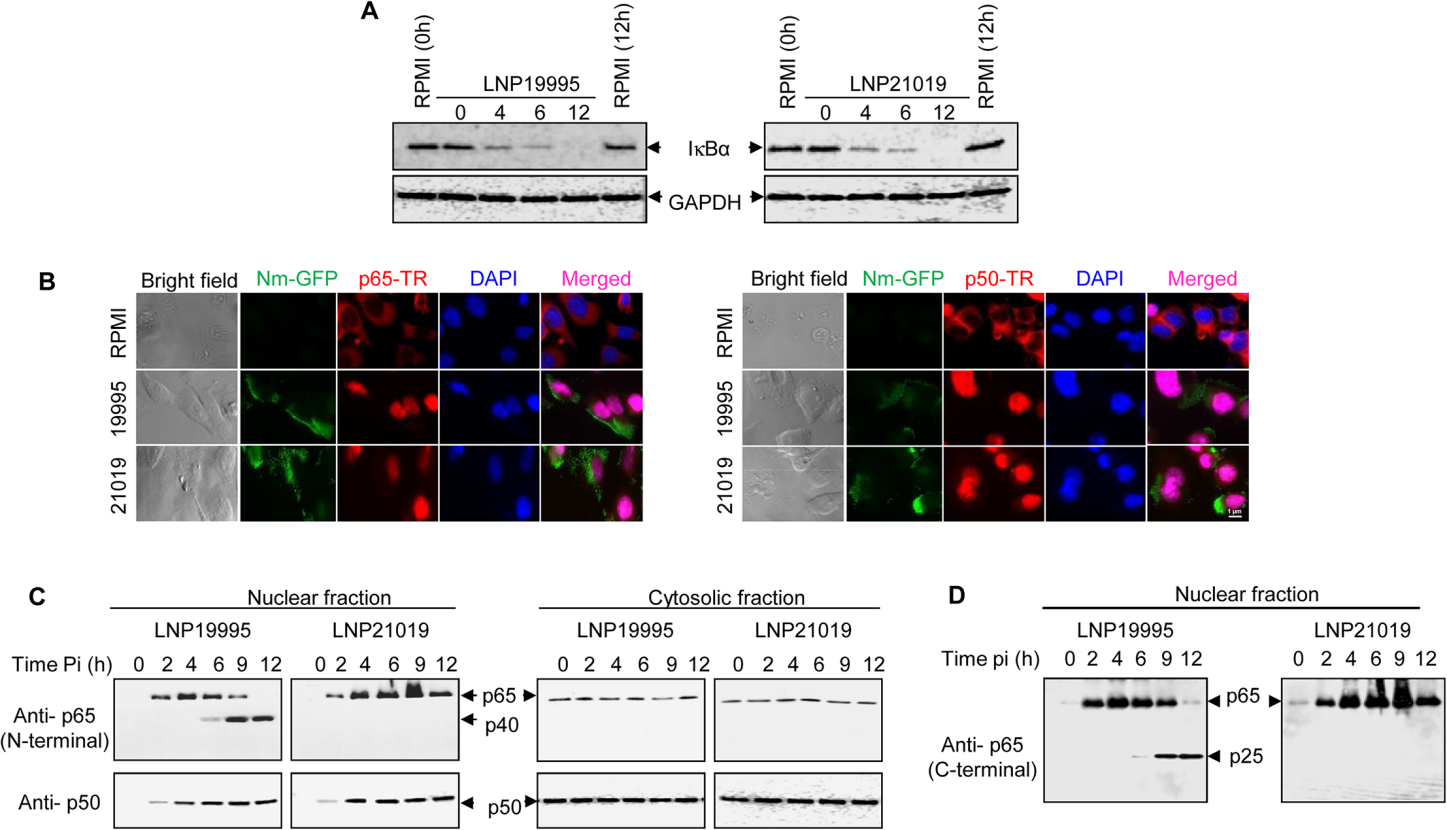
Meningococcal ST-11 isolates promote nuclear cleavage of p65 at late steps of infection. (A) Meningococcal infection is accompanied by IkBα degradation. Hec-1-B epithelial cells were infected with LNP19995 (ST-11) or LNP21019 (non-ST-11) isolates for the indicated time points or left uninfected. Cytosolic fractions were subjected to immunoblotting analysis for I-κBα. Immunoblotting with anti-GAPDH antibodies was used as a protein loading control. (B) Nuclear translocation of both p65/RelA and p50/NF-κB1 subunits was not altered by meningococcal infection. Hec-1B cells were infected with GFP-expressing LNP19995 or LNP21019 or left non-infected (RPMI). After 9h, cells were fixed with 3.7% PFA, permeabilised and probed with mouse anti-p65/RelA mAb (left panel) or rabbit anti-p50/NF-κB1 (right panel) and Texas red (TR)-conjugated appropriate secondary antibody. Nuclei were stained with DAPI. Fluorescence was analyzed using immuno-fluorescence microscopy. Scale bar (1 μm) is shown. Data are representative of three independent experiments. (C) Nuclear or cytosolic fractions from (A) were analysed by immunoblotting using anti-N-terminal p65/RelA specific mAb (upper panels) or anti-p50/NF-κB1 rabbit polyclonal antibody (lower panels). Shown is a representative blot of three independent experiments. p65/RelA cleavage product p40 is indicated by arrows. (D) The blot of nuclear fractions from (C) was stripped and probed against a goat polyclonal antibody directed against C-terminal region of p65/RelA. The cleavage product p25 is indicated by arrowhead.

Immunoblotting analysis showed that both ST-11 and non-ST-11 isolates caused IκBα degradation (Figs 1A and S1A). Consistently, nuclear translocation of both p65/RelA and p50/NF-κB1 subunits was detected by immunofluorescence microscopy and immunoblotting (Fig 1B and 1C). While persisting in the nuclear fraction of cells infected with the non-ST-11 isolate LNP21019, the level of p65/RelA decreased beyond 6 h in the nuclear fraction of cells infected with the non-ST-11 isolate LNP19995. Intriguingly, the decrease in the nuclear amount of p65/RelA subunit paralleled with a progressive increase of a 40 kDa reactive band (named p40) that was recognized with the same antibody (Fig 1C, left panel). The p40 band could result from the cleavage of p65/RelA subunit. Indeed, enhanced detection of a ~25-kDa reactive band (named p25) with a C terminus-specific antibody to p65/RelA consolidated this hypothesis (Fig 1D). Similar results were obtained with other ST-11 and non-ST-11 isolates (S1B Fig) and with A549 airway epithelial cell line (S1C Fig). Cleavage of p65/RelA was restricted to the nuclear fraction as no alteration of p65/RelA was observed in cytosolic fractions (Fig 1C, right panel). It is worth noting that alteration of NF-κB was selective to p65/RelA subunit because expression of the p50/NF-κB1 subunit was not affected by meningococcal infection in either fraction (Fig 1C, bottom blots). Taken together, these results suggest that ST-11 isolates promote nuclear cleavage of p65/RelA subunit during late steps of infection.

### A secreted serine protease is involved in the nuclear cleavage of p65/RelA

**Fig 2:**
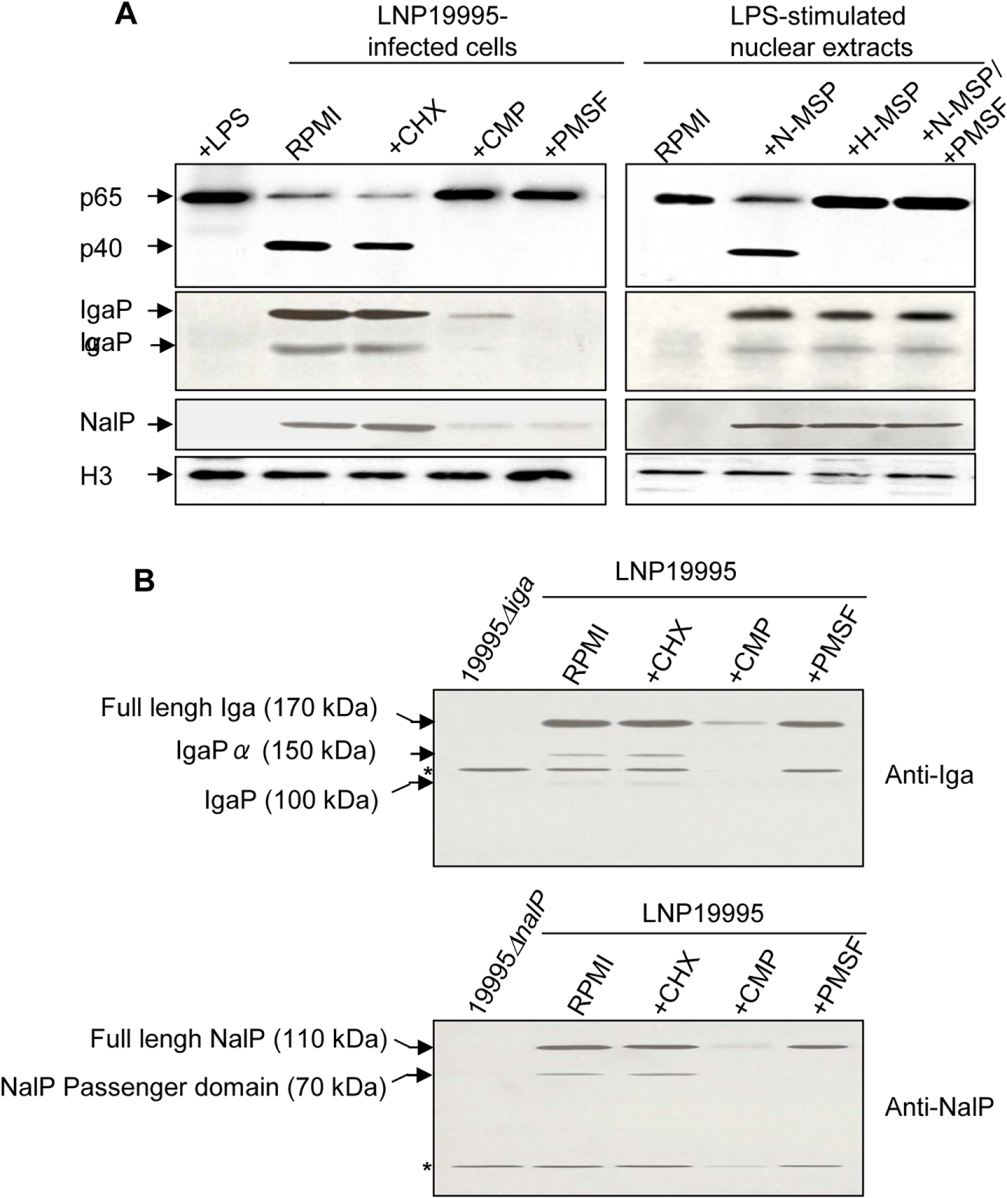
Nuclear cleavage of p65/RelA is carried out by a ST-11 meningococcal secreted serine protease. (A) For in vivo experiments (left panel), Hec-1B cells were infected for 9h in absence or presence of 1 μg.ml^-1^ cycloheximide (CHX), 5μg.ml^-1^ chloramphenicol (CMP) or 5 mM PMSF. After incubation cells were harvested, and nuclear fractions were prepared. For *in vitro* experiments (right panel), nuclear extracts were prepared from LPS-stimulated cells and then incubated with native meningococcal secreted proteins (N-MSP) in absence or presence of 5 mM PMSF, or heat-inactivated MSP (H-MSP) of the ST-11 isolate LNP19995. Samples were resolved by SDS-PAGE and immunoblotted with anti-p65/RelA mAb, anti-IgA protease or anti-NalP sera.Histone H3 expression (lower panel) was used as loading control. Immunoblot is representative of three independent experiments which yielded similar results. (B) Whole lysates of bacteria recovered from (A), were resolved by SDS-PAGE and immunoblotted with anti-IgA protease or anti-NalP sera.Mutant strains 19995*Δiga* and 19995*ΔnalP* were used as negative controls. Full length precursors and autocleaved forms are indicated by arrows. * represent a cross reactive bands.

**Fig 3:**
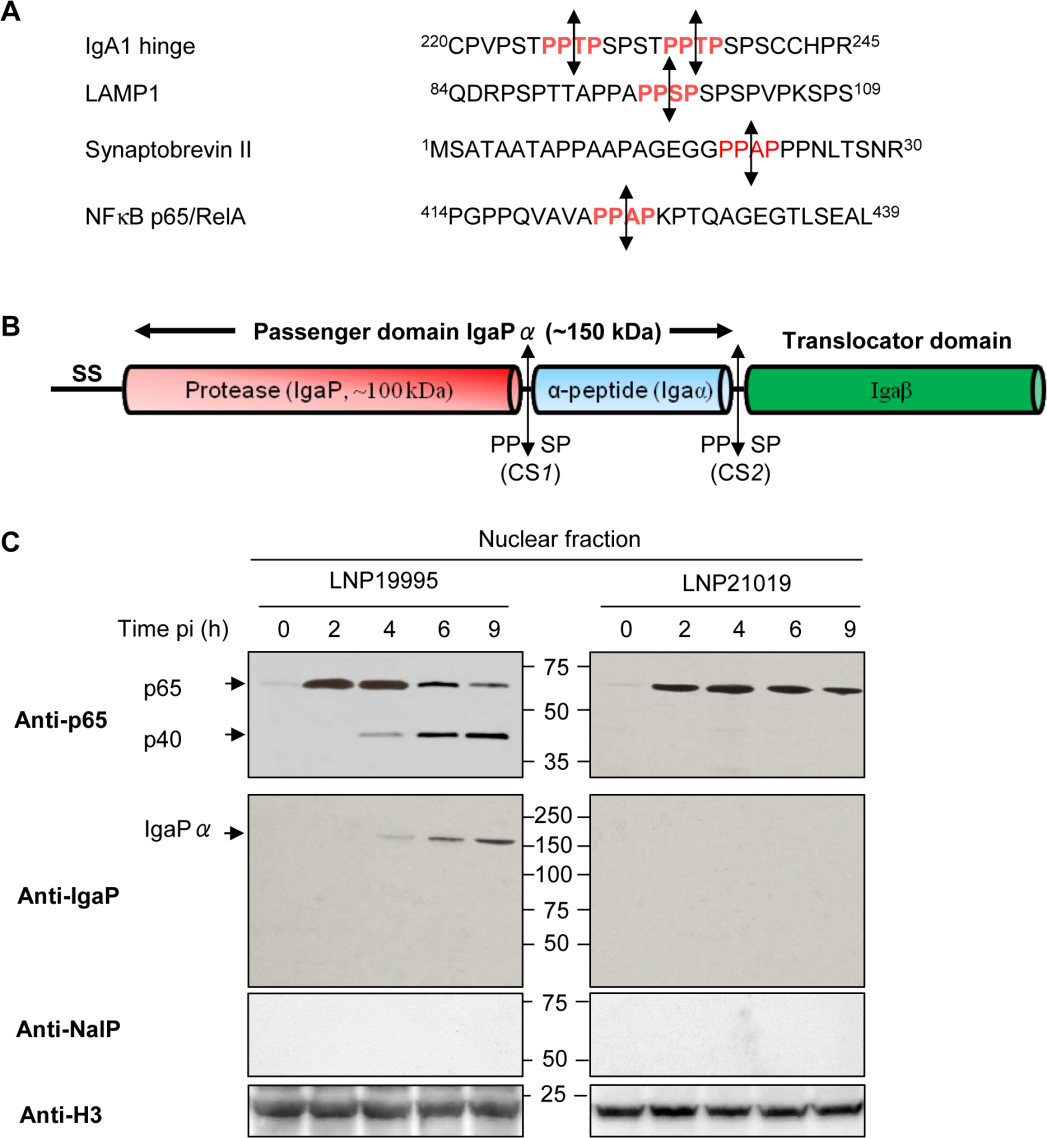
Cleavage of p65/RelA correlates with nuclear translocation of IgA protease. (A) Similarity between amino acid sequences of the hinge region of human IgA1, part of the N-terminal peptide of the human lysosome-associated membrane glycoprotein 1 LAMP1 (Accession N°: AAH06345), synaptobrevin II (Accession N°: AAF15551) targeted by neisseria IgA protease and part of the carboxy terminal peptide of the p65/RelA subunit of NF-κB (Accession N°: CAA80524). The position of amino acid residues are indicated by superscript numbers delimiting each peptide sequence. Specific cleavage sites are indicated in red and the cleavage position is represented by double-headed arrows. (B) Schematic overview of the various domains and sub-domains of neisserial IgA protease. Autocatalyic cleavage sites CS1 and CS2 and their sequences are indicated by double-headed arrows. SS: signal sequence. (C) Nuclear fractions were prepared from Hec-1-B cells infected with LNP19995 or LNP21019 at indicated time points. Samples were resolved in SDS-PAGE and probed with anti-N-terminal p65 mAb or rabbit polyclonal serum specific to IgaP sub-domain or anti-NalP specific serum. Histone H3 was used as loading control.

We next showed that cleavage of p65/RelA was blocked when Hec-1-B cells were infected with LNP19995 (ST-11) in presence of 5μg.ml^-1^ chloramphenicol antibiotic that inhibits bacterial protein synthesis but not by cycloheximide (a eukaryotic protein synthesis inhibitor), suggesting that cleavage of p65/RelA required de novo synthesized bacterial proteins (Fig 2A, left panel). Moreover, cleavage of p65/RelA was observed upon incubation of the nuclear fraction from LPS-stimulated cells with native (but not heat-inactivated) bacterial culture supernatant of LNP19995 suggesting that meningococcal secreted proteins (MSP) may be involved (Fig 2A, right panel). The presence of the serine proteases inhibitor PMSF abrogated the cleavage of p65/RelA in both, *in vivo* and *in vitro* cleavage assays (Fig 2A) further suggesting that the involved secreted meningococcal protein is most likely a serine protease that is able to reach the nuclear compartment of infected cells. We therefore prepared intact cytosolic-free nuclei from infected or uninfected cells (see Materials and Methods section and S2A Fig). Proteins of isolated nuclei were then subjected to 2D-gel electrophoresis and immnublotting with anti-MSP specific mouse serum. Several spots of high molecular weight reacted with the serum from infected but not from non-infected cells (S2B Fig, upper panel, compare *a* and *c*). None of these spots reacted with the pre-immune serum (S2B Fig, lower panel). The apparent electrophoretic mobility and isoelectric point of one of these spots is compatible with the neisserial IgA protease, a type V autotransporter secreted serine peptidase (S2B Fig, upper panel, compare *b* and *d*) [22]. Moreover, examination of the primary structure of p65/RelA revealed the presence of a specific IgA protease cleavage site close to the C terminus of p65/RelA subunit P-P-|-X-P (shown by the vertical line), where X is either S, T or A amino-acid (Fig 3A) [23,24,25].

### Nuclear cleavage of p65/RelA by ST-11 isolates correlates with the nuclear accumulation of α-peptide linked-IgA protease

The meningococcal IgA protease is expressed as a polyprotein precursor molecule having three domains (Fig 3B). These include an N-terminal signal sequence, a C-terminal outer membrane-embedded translocator domain (Iga*β*) and the central secreted passenger domain (IgaPα) that includes the protease sub-domain (referred to as IgaP) followed by α-peptide sub-domain (referred to as Iga*α*) [26,27,28] harbouring eukaryotic nuclear localisation signal (NLS) [29]. A cleavage site CS1 upstream Igaα resulted in release of the IgaP 100 kDa fragment. In presence of NalP (a phase variable meningococcal surface protease), an alternative proteolysis at cleavage site CS2 downstream Igaα, allowed the release of a predominant 150 kDa form corresponding to IgaP linked to Igaα (referred to as IgaP*α*) (Fig 3B) [28,30]. Both of these two proteins (100 kDa and 150 kDa) are detected in MSP of meningococcal isolates with a “phase on” *nalP* gene (S3A Fig). The same pattern was observed in the cytosolic fraction of infected cells (S3A Fig, lower left panel). Interestingly, the anti-IgaP polyclonal serum detected a progressive accumulation of IgaPα, but not IgaP, in the nuclear fraction of cells infected with ST-11 isolates that coincided with the cleavage of p65/RelA. However, the absence of IgA protease from the nucleoplasm of cells infected with non-ST-11 isolates correlated with the expression of intact p65/RelA (Figs 3C and S3B). Collectively, these results suggest that nuclear cleavage of p65/RelA in cells infected with ST-11 isolates correlated with the nuclear appearance of IgaPα.

### IgA protease of ST-11 isolates mediates nuclear cleavage of p65/RelA

**Fig 4:**
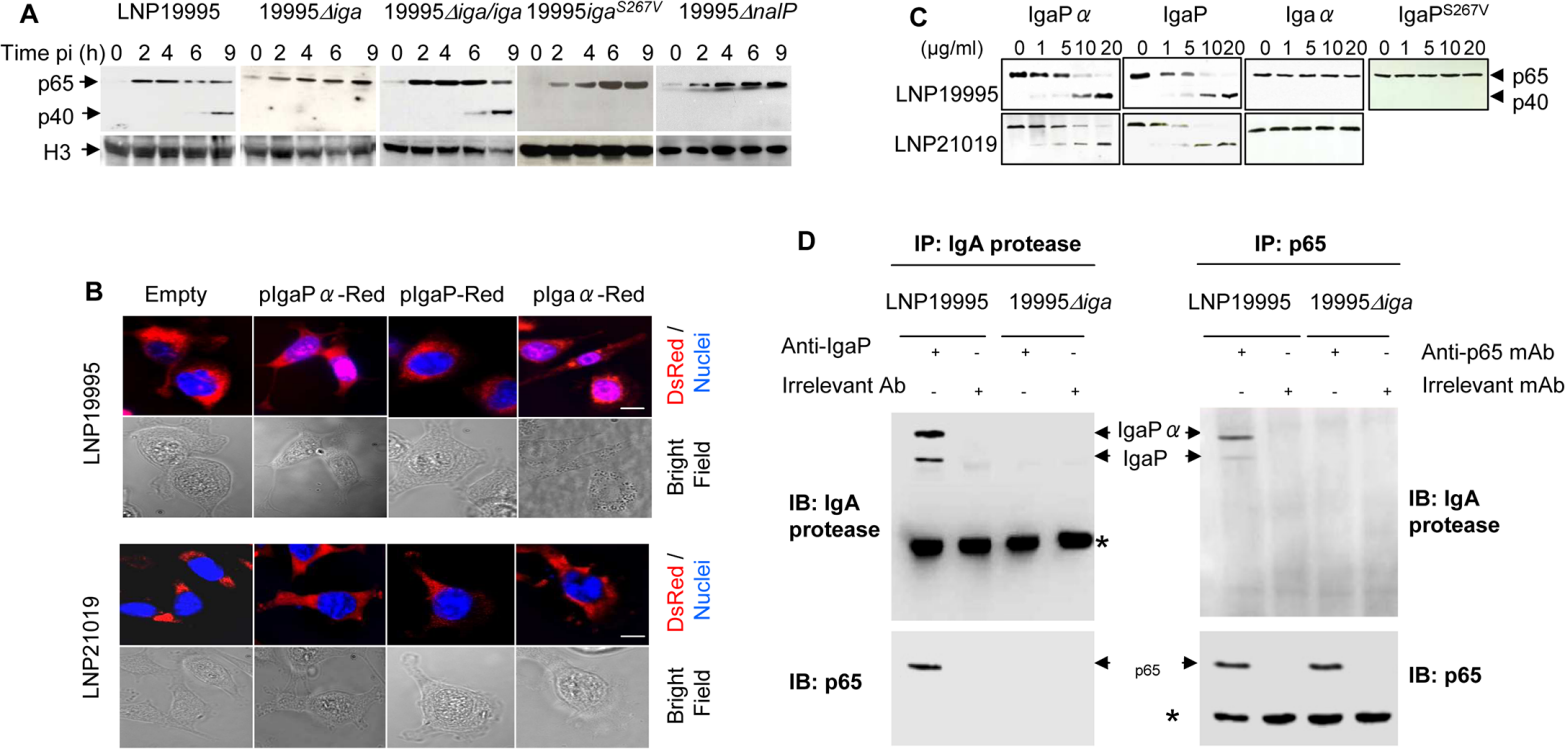
IgA protease of ST-11 isolates interacts with and mediates nuclear cleavage of p65/RelA. (A) Hec-1-B cells were infected with the indicated strains. For each time point, nuclear fractions were prepared and analysed by immunoblotting using anti-p65 mAb. Histone H3 was used as loading control. (B) Localization of IgA protease sub-domains in transfected infected cells. IgaP, Igaα or IgaPα of LNP19995 (upper panel) or LNP21019 (lower panel) were fused to DsRed. Hec-1-B cells were then transfected with either construct or pDsRedN1 plasmid (Empty vector). After 48 hours, cells were washed, fixed with 3.7% PFA and stained with DAPI (blue) before visualisation under the microscope. Scale bar (10 μm) is shown. (C) *In vitro* activity of purified IgA protease passenger sub-domains against p65. IgaP, Igaα or IgaPα or IgaP^S267V^ of the strains LNP19995 (upper panel) and LNP21019 (lower panel) were subcloned and purified as C-terminal His_6_-tagged proteins. Nuclear extracts (1 μg) from LPS-stimulated Hec-1-B cells were mixed with the indicated concentrations of the purified proteins. The reaction mixtures were incubated at 25°C for 3 hours and then analyzed with antibodies against p65/RelA (N-terminal specific). (D) IgA protease interacts with p65/RelA. Nuclear extracts from LPS-stimulated Hec-1-B cells were mixed with MSP prepared from LNP19995 or 19995*Δiga* for 6h in presence of 5 mM PMSF. The samples were immunoprecipitated with anti-IgaP specific serum or irrelevant rabbit Ab (left panel) or anti-p65/RelA mAb or irrelevant mAb (right panel) and analyzed by immunoblotting with antibodies against p65/RelA and IgaP, respectively. * indicates antibody heavy chain.

To establish a direct cause effect relationship between IgA protease expression of ST-11 isolates and the nuclear cleavage of p65/RelA, we generated an *iga* deletion mutant (named 19995*Δiga*) and the complementing strain (named 19995*Δiga/iga*). The absence of IgaP and IgaPα expression was confirmed in the MSP fractions prepared from 19995*Δiga* mutant strain, while the expression of both IgA protease forms was restored in the MSP of the complementing strain as the parental strain (S4A Fig). Furthermore, substitution of *iga* by the spectinomycin resistance cassette in the mutant 19995*Δiga* had no polar effect on the expression of *trpB* gene downstream *iga* as confirmed by RT-PCR (S4B Fig). Hec-1-B cells were infected with either strain and expression of p65/RelA in the nuclear compartment was followed by immunoblotting. Nuclear cleavage of p65/RelA occurred progressively beyond 6h in LNP19995-infected cells, but not in the 19995*Δiga*-infected cells (Fig 4A). Consistently, NF-κB transcriptional activity was altered in cells infected with LNP19995 whereas sustained in cells infected with 19995*Δiga* as the non-ST-11 isolate LNP21019 (S4C Fig). The complemented strain 19995*Δiga*/*iga*_*19995*_ restored the cleavage of p65/RelA in infected cells resulting in alteration of NF-κB activity comparably to the WT strain (Figs 4A and S4C).

van Ulsen *et al*., reported that the serine protease autotransporter protein NalP is involved in modulating the processing of other meningococcal autotransporters, including IgA protease. As reported elsewhere, the *nalP* mutant strain 19995*ΔnalP*, secreted increased level of IgaP lacking the α peptide and strongly decreased level of IgaPα comparing to the WT parental strain LNP19995 (S4D Fig and [30]). Consistently, 19995*ΔnalP* strain reproduced similar phenotypes as 19995*Δiga* mutant regarding cleavage of p65/RelA (Fig 4A). It is worth noting that NalP was not detected in the nuclear fraction of LNP19995-infected cells (Fig 3C), ruling out any direct effect of NalP on the nuclear cleavage of p65/RelA. These results suggest that NalP is involved in down regulation of NF-κB activity by ST-11 isolates, most likely through modulation of IgaPα secretion. To further determine the role of α-peptide in the nuclear translocation of ST-11 IgA protease, Hec-1-B cells were transfected separately with mammalian C-terminus DsRed-tagged constructs harbouring either IgaP_19995_, Igaα_19995_, or IgaPα_19995_. We found that IgaP_19995_, as DsRed control empty vector, was located in the cytosol of transfected cells, whereas a substantial amount of DsRed signal was located into the nucleus of cells transfected with Igaα_19995_ or IgaPα_19995_ (Fig 4B, upper panel).

**Fig 5:**
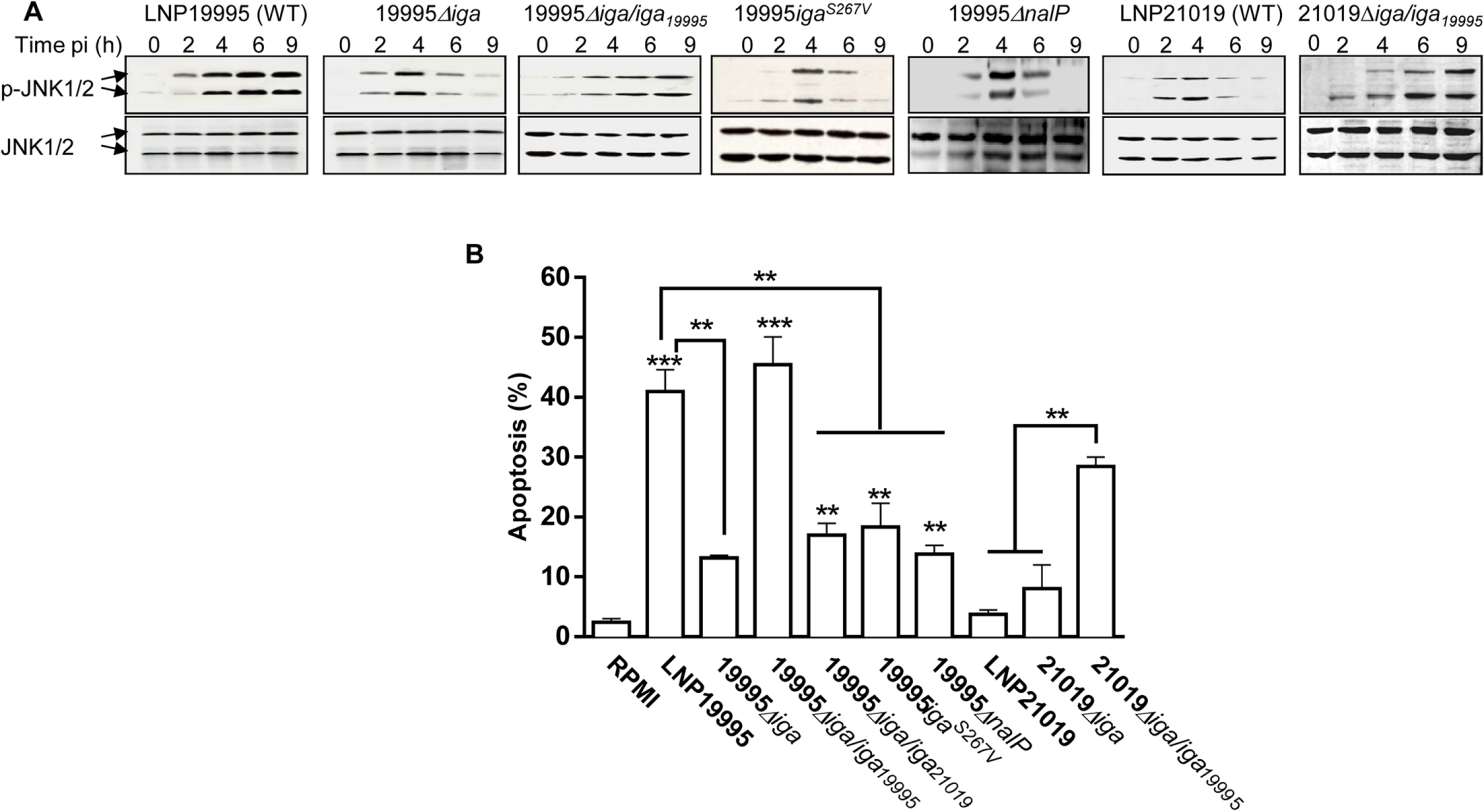
IgA protease of ST-11 isolates promotes sustained activation of JNK and apoptosis of epithelial cells. (A) Hec-1B cells were infected with the indicated strains. At each time point, samples were lysed and immunoblotted with rabbit polyclonal anti-phospho-JNK antibodies (upper panel). The membranes were subsequently stripped and re-probed with rabbit polyclonal anti-JNK antibodies as protein loading controls. Blots are representative of three separate experiments with similar results. (B) Cells were left uninfected or infected with the indicated strains for 9 h then analysed for apoptosis using annexin V staining and flow cytometry. Histogram bars represent the mean ± SD from three independent experiments. **, *P* < 0.01, ***, *P* <0.001.

Accordingly, purified His6-tagged catalytic protease sub-domain (IgaP_19995_) or full passenger domain (IgaPα_19995_) (S5A Fig) were able to provoke cleavage of p65/RelA when incubated with nuclear fractions prepared from LPS-stimulated Hec-1-B cells (Fig 4C, upper panel). However, purified His6-tagged α-peptide sub-domain (Igaα_19995_) (S5 Fig) was unable to do so (Fig 4C, upper panel). The role of the protease sub-domain in the cleavage of p65/RelA would imply that the active site IgA protease is required for the processing of p65/RelA. To test this possibility, we generated an active-site mutant strain 19995*iga* ^*S267V*^. Immunoblot examination was performed to verify the expression of Iga^S267V^. As expected, disabled active-site resulted in accumulation of full-length Iga^S267V^ in the whole cell lysate (S4D Fig), hence 19995*iga* ^S267V^-infected cells expressed intact nuclear p65/RelA (Fig 4A) comparably to 19995*Δiga* mutant strain. Furthermore, purified IgaP-S^267^V (Fig 5B) was unable to cleave p65/RelA *in vitro* in contrast to the WT IgaP (Fig 4C). These results corroborate with the effect of PMSF observed in Fig 2A. Indeed, PMSF treatment strongly decreased the levels of secreted IgA protease and other serine proteases, including NalP in the culture medium of infected cells (Fig 2A). This effect did not result from a defect of protein expression, as immature full length serine proteases were detected in the bacterial lysates recovered from the same culture medium (Fig 2B). Altogether, these set of experiments suggest that active site of IgA protease is required for its role in processing p65/ReA. The cleavage of p65/RelA seems to occur through a direct interaction with IgA protease as suggested by co-immunoprecipitation experiments using MSP prepared from WT, but not 19995*Δiga* mutant, when pull-down was performed with an anti-IgaP specific serum (Fig 4D, left panel). Both IgaP and IgaPα, co-immunoprecipitated with p65/RelA when pull-down was performed with anti-p65 mAb comparing to irrelevant mouse mAb (Fig 4D, right panel).

### IgA protease of ST-11 isolates contributes to sustained activation of JNK and apoptosis of epithelial cells

We next examined the role of IgA protease in inducing apoptosis of epithelial cells upon infection of cells with the parental WT LNP19995 or with the isogenic strains 19995*Δiga* and 19995*Δiga/iga*_19995._ The activation of JNK and apoptotic levels were analysed over time using immunoblotting and flow cytometry, respectively. The mutant strain 19995*Δiga* promoted a transient activation of JNK (Fig 5A) and a significant decrease in apoptotic level of epithelial cells(Fig 5B) comparing to the parental strain. Consistently, the isogenic mutants 19995*iga* ^*S267V*^ (disabled in S267 active site) and 19995*ΔnalP* (that was affected in secretion of IgaPα) reproduced similar phenotypes as 19995*Δiga* (Fig 5). The complemented strain 19995*Δiga/iga*_*19995*_ restored all these phenotypes similarly to the WT strain (Fig 5), suggesting that IgA protease of ST-11 isolates contribute to apoptosis of epithelial cells.

### Non-ST-11 isolates release α-peptide lacking IgA protease defective in targeting nuclear compartment

**Fig 6:**
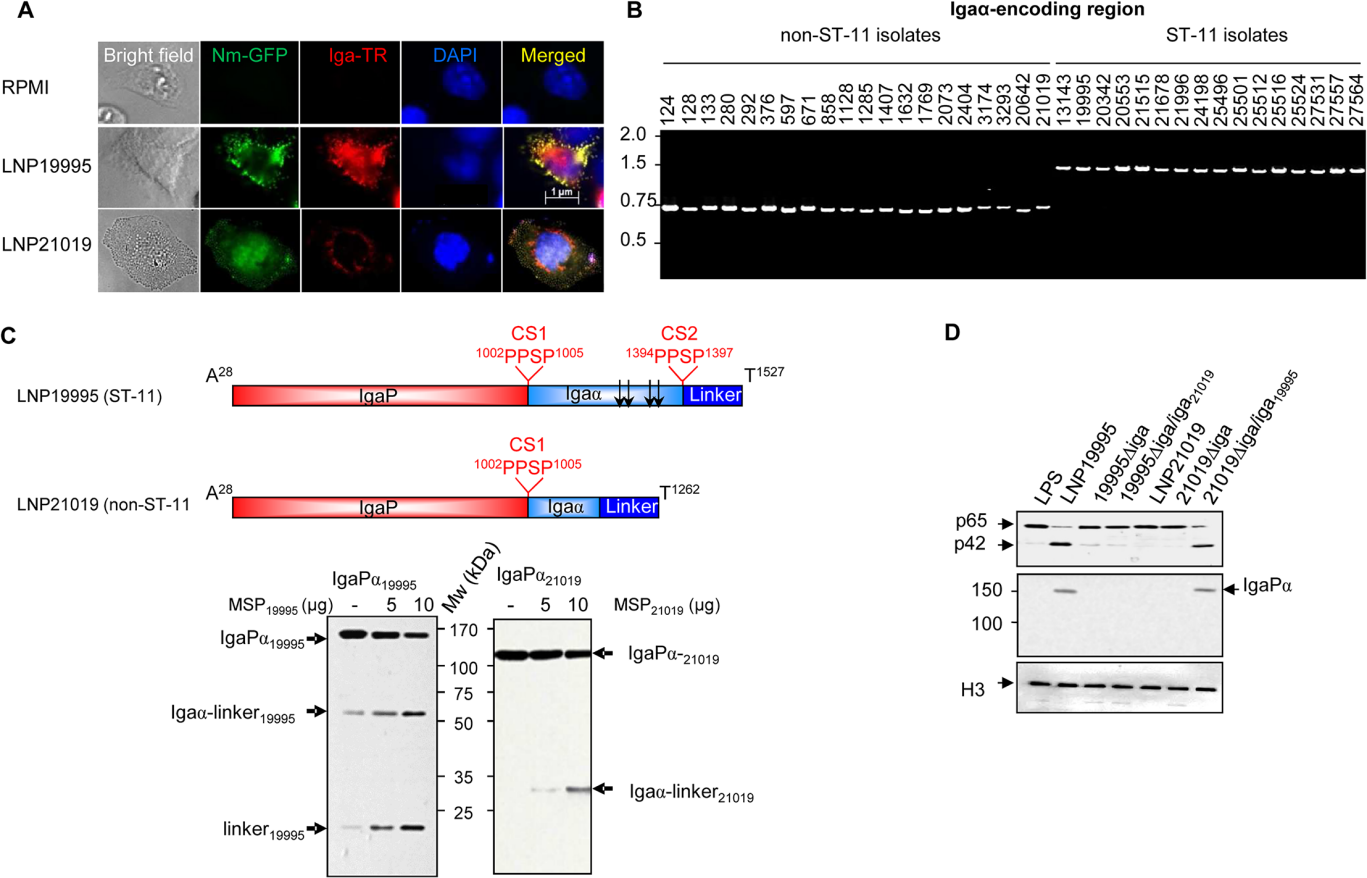
Non-ST-11 isolates release α-peptide-lacking IgA protease. (A) Subcellular localisation of IgA protease in infected cells. Hec-1B cells were infected with GFP-expressing LNP19995 or LNP21019 (green) or left uninfected. After 12 h of infection, cells were fixed with 4% PFA, permeabilised and stained with anti-IgaP polyclonal serum and Texas Red (TR)-conjugated anti-rabbit IgG (red). Nuclei were stained with DAPI. Fluorescence was analyzed using immunofluorescence microscopy. Data are representative of three independent experiments. (B) Analysis of ST-11 and non-ST-11 isolates by PCR using primers specific to α-peptide sub-domain. PCR products were amplified from genomic DNA of strains indicated above the gel, using the couple of primers alphaFwNhe / alphaRevSma. PCR products were separated by electrophoresis in 1% agarose gels and stained with ethidium bromide. PCR amplification generated amplicons of ~ 1500 bp in all ST-11 isolates, while amplicon sizes ranged between 687 and 770 bp in non-ST-11 isolates. Molecular sizes (kb) are indicated in the left side. (C) Upper panel. Non scaled schematic representation of the passenger subdomains of meningococcal IgA protease from isolates LNP19995 (ST-11) and LNP21019 (non-ST-11). The positions of autocatalytic processing sites and their sequences (PPSP) are indicated. Arrows indicate positions of NLSs in α-peptide of the ST-11 isolate. Lower panel. Five hundred nanograms of the C-terminal His_6_-tagged passenger domain of the strains LNP19995 (IgaPα_19995_) or LNP21019 (IgaPα_21019_) were mixed with 5 or 10 μg of MSPs. After 3 h, the reaction mixtures were analyzed with immunoblot using anti-His tag mAb. The different cleavage products are indicated by arrows. Mw indicates the molecular weight. (D) IgA protease of ST-11 isolates restores the capacity of non-ST-11 isolates in cleaving nuclear p65. Each of the 19995*Δiga* and 21019*Δiga* were complemented with the heterologous *iga* allele of the WT strain LNP21019 and LNP19995, respectively. Hec-1-B cells were infected for 12 h with the parental WT strains, the isogenic *iga* knock-out mutant strains or the heterologous complemented strains. Nuclear fractions prepared from infected cells, were resolved by SDS-PAGE and were probed with anti-p65 mAb (N-terminal specific) or polyclonal serum anti-IgaP. LPS-treated cells were used as positive control for nuclear translocation of NF-κB. Immunoblot with anti histone H3 was used as loading control.

Cells exhibited sustained activation of NF-κB when infected by the non-ST-11 strain LNP21019 with a transient activation of JNK and apoptotic level close to uninfected cells (LNP21019 in Figs 5A, 5B and S4C). To understand why non-ST-11 isolates did not alter the nuclear activity of p65/RelA *in vivo*, we examined the expression and subcellular localisation of IgA protease of these isolates. Unlike ST-11 isolates, only the 100 kDa-IgaP was detected in the MSP fractions prepared from non-ST-11 isolates that could be also found in the cytosolic fractions of infected cells (S3A Fig, right panel), suggesting that non-ST-11 isolates were unable to release IgaP linked to α-peptide, although *a priori* express NalP (S3A Fig). These findings were also confirmed by immunofluorescence microscopy examination. Indeed, after 12 h of infection, the fluorescent signal specific to IgA protease (red) appeared in the cytosolic compartment but a substantial signal colocalized also with the nuclear compartment (blue) of cells infected with the ST-11 isolate LNP19995 (appears as magenta in merged panel). In contrast, IgA protease of the non-ST-11 isolate LNP21019 appeared as a punctuate pattern exclusively localized in the cytosolic space (Fig 6A). These results suggest that IgA protease released from non-ST-11 isolates did not present a defect in cell internalisation but rather in nuclear translocation. It is worth noting that purified IgaP sub-domain of the non-ST-11 isolate LNP21019 (IgaP_21019_), as MSP prepared from different non-ST-11 isolates, exhibited an *in vitro* cleavage activity of p65/RelA comparable to ST-11 isolates (Fig 4C, lower panel), ruling out a defect in the protease activity. We then compared α-peptide encoding regions of non-ST-11 isolates to those from ST-11 isolates. From all ST-11 isolates examined, PCR amplification yielded a single PCR band of ~ 1500 bp (Fig 6B). Amino acid-derived sequence analysis revealed the presence of two bipartite NLS sequences flanked by two cleavage sites CS1 and CS2 (Figs 6C and S6). Of the 20 non-ST-11 isolates examined, all yielded a single α-peptide PCR product with a smaller length than those resulted from ST-11 isolates (Fig 6B). This difference resulted from the deletion of ∼813 bp (271 amino-acids) encompassing the NLS sequences (S6 Fig). Indeed, DsRed fused to α-peptide of the carriage isolate remained in the cytosolic compartment (Fig 4B, lower panel) suggesting impaired nuclear transport. Importantly, all isolates shared the cleavage site CS1 separating IgaP and α-peptide (Igα). However, the alternative cleavage site CS2 downstream α-peptide was missing in all non-ST-11 isolates (S6 Fig). These observations were confirmed experimentally by incubating the purified C-terminal His_6_-tagged passenger domains IgaPα of LNP19995 (1500 residues extending from A^28^ to T^1527^) and LNP21019 (1235 residues extending from A^28^ to T^1262^) with the MSPs of LNP19995 and LNP21019, respectively (Fig 6C). Cleavage of LNP19995 passenger domain generated two His_6_-tagged fragments of ~ 60 kDa (that resulted from cleavage at the site CS1) and ~14 kDa (that resulted from the cleavage at site CS2) in presence of increasing amounts of MSPs. These fragments correspond to Igaα (~44–45 kDa) associated to the linker domain (~14 kDa); and the linker domain alone, respectively (Fig 6C, left panel). However, only one His_6_-tagged fragment of ~ 31 kDa (corresponding to Igaα associated to the linker) was generated from the cleavage at CS1 of LNP21019 passenger domain (Fig 6C, right panel). These results suggest that proteolytic cleavage of IgA protease may therefore occur at two cleavage sites in ST-11 isolates LNP19995: CS1 autocleavage site (separating the protease domain from the α peptide) and CS2 autocleavage site (separating the α peptide from the translocator domain). However, the proteolytic cleavage may occur at the unique CS1 site, leading to release of α-peptide-lacking IgA protease in non-ST-11 isolates.

To examine more thoroughly the role of α-peptide, each of the *iga* knock-out mutant strains 21019*Δiga* and 19995*Δiga* were complemented with *iga* allele of the heterologous isolate LNP19995 and LNP21019, respectively. The resulting recombinant strains 21019*Δiga/iga*_*19995*_ and 19995*Δiga/iga*_*21019*_ released comparable IgA protease pattern as the non-ST-11 isolate LNP19995 and the non-ST-11 isolate LNP21019, respectively (S4A Fig). More importantly, the recombinant 21019*Δiga/iga*_*19995*_ altered full length p65/RelA, promoted sustained activation of JNK and promoted substantial apoptotic level, concomitantly to the accumulation of the IgaPα in the nuclear compartment of infected cells comparably to ST-11 isolates (Figs 5 and 6D). In contrast, the recombinant strain 19995*Δiga/iga*_*21019*_ did not promote nuclear localisation of IgaPα, or alteration of the full length p65/RelA in the nuclear compartment of infected cells (Figs 5B and 6D). These results suggest that IgA protease of ST-11 isolates may restore the ability of non-ST-11 isolates to cleave the nuclear p65/RelA and promote apoptosis of epithelial cells.

### ST-11 IgA protease-mediated cleavage of p65/RelA leads to alteration of selective NF-κB-responsive targets

**Fig 7:**
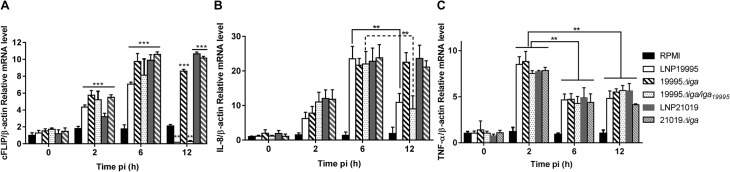
ST-11 IgA protease-mediated nuclear cleavage of p65/RelA alters selectively expression of NF-κB responsive genes. Hec-1-B cells were infected for the indicated periods, After infection of Hec-1-B cells with the indicated strains and periods, mRNA levels of c-FLIP (A), IL-8 (B) and TNF-α (C) were evaluated by real-time PCR. Target mRNA expression was normalized to β-actin mRNA. ***, *P*<0.001; **, *P*<0.01

To test for the functional relevance of ST-11 IgA protease-mediated nuclear cleavage of p65/RelA, we asked whether ST-11 IgA protease-mediated cleavage of p65/RelA culminates in alteration of the expression of NF-κB responsive genes. For that purpose, relative mRNA levels of three NF-κB responsive targets; the pro-inflammatory cytokines TNF-α, interleukin (IL)-8 and the major anti-apoptotic protein cFLIP [31] were examined by qRT-PCR. As shown in Fig 7A and 7B, expression of both IL-8 and cFLIP increased progressively and reached a peak level at 6h post infection. While persisted longer in cells infected with the carriage isolate LNP21019 and the *Δiga* mutants, mRNA levels of cFLIP and IL-8 decreased significantly in cells infected with the ST-11 isolate LNP19995 and the complemented strain 19995*Δiga/iga* comparing to the isogenic mutant strain 19995*Δiga*, although more pronounced for cFLIP than for IL-8 which remained detectable at substantial level. Interestingly, peak levels of TNF-α transcripts occurred as early as 2h post-infection, after which they rapidly decreased by 6h and remained substantially stable up to 12h. The expression profile of TNF-α was comparable between WT and *Δiga* mutants in contrast to the former targets (Fig 7C). Taken together, these results suggest that cleavage of p65/RelA mediated by ST-11 IgA protease resulted in alteration of selective NF-κB-responsive targets.

## Discussion

Several pathogens elicit apoptotic cell death by inducing an inflammatory response that may lead to disruption of tissue barriers allowing efficient microbial spread in the host [32,33]. In this work, we revealed a mechanism used by ST-11 isolates of Nm (but not by the non-ST-11 isolates) to disrupt the epithelial cells by apoptosis induction. The secreted IgA protease from these isolates is transported to the nucleus of infected cells where it cleaves the p65/RelA component of NF-κB complex. NF-κB is tightly controlled by negative feedback loops mediated primarily by IκB, leading to oscillatory responses, in which NF-κB shuttles between the cytoplasm and nucleus with the period of about 100 min [20]. Comparing to other reports [34], the steady decrease of IκB over the period of 12h of infection was surprising. Meningococcal infection may interact with other components of NF-κB signaling pathway including IKK complex and/or A20/TNFAIP3 to attenuate the negative feedback loop mediated by IκB [35,36,37,38] although cell lines and bacterial strains used in this study may account for these differences. Persistent degradation of IκB has also been reported for other bacterial pathogens, but the mechanism responsible for that decrease remains unknown [39,40,41,42]. Additional research will clearly be needed to elucidate the precise mechanism of this phenomenon. On the other hand, aberrantly prolonged NF-κB activation may have deleterious cellular effects [43,44,45,46,47]. However, the persistent NF-κB activation observed in cells infected with non-ST-11 isolates suggest that these isolates drive somehow mechanism(s) required to maintain a fine balance of NF-κB activation in infected cells to dampen the inflammatory response at levels that are advantageous for bacteria yet that would allow the maintenance of a healthy ecological niche.

The expression of NF-κB-regulated genes was altered as a consequence of ST-11 IgA protease-mediated cleavage of p65/RelA. Interestingly, this alteration targets selectively some NF-κB-regulated genes (for instance IL-8 and cFLIP). In contrast to TNF-α, expression of IL-8 and cFLIP, follows obviously the kinetic of NF-κB activity (S4C Fig). These differential effects of IgA protease could be related to the balance between the level of available intra-nuclear IgA protease and the kinetic of expression for a given NF-κB-regulated target. Indeed, TNF-α is an early up-regulated cytokine that is rapidly down-regulated within 4 to 6h post-infection, a time for which the level of IgA protease may not be good enough to compromise NF-κB activity and hence TNF-α expression. Alternatively, IgA protease may interact with NF-κB in selective manner dependent on the nature of NF-κB-regulated promoter to alter their expression following cleavage of NF-κB p65 subunit. This hypothesis suggests that α peptide-associated IgA protease may have a DNA-binding activity to some NF-κB-responsive promoters. In agreement with this hypothesis, Arenas *et al*. [48] have recently reported the ability of α peptide subdomain to bind DNA. Alteration of IL-8 and TNF-α expression was not completely compromised, in contrast a substantial levelq of IL-8 and TNF-α mRNAs remained detected over 12 h of infection. This may be related to an alternative mechanism regulating the expression of these targets independently to NF-κB. Indeed, the role of JNK in up-regulation of IL-8 has been demonstrated [49,50,51].

Alteration of NF-κB activity promoted by ST-11 isolates should have multi-faceted consequences. For instance, activation of JNK was sustained in ST-11-infected cells and this may account for the high level of IL-8 expression despite alteration of NF-κB activity. It has been reported that transient activation of JNK is normally terminated by MAP Kinase phosphatases (MKPs), a mechanism itself controlled by NF-κB survival signaling [52]. Alteration of NF-κB may therefore down regulate the MKPs activity and provoke persistent JNK activation that may account for high levels of IL-8 expression in one hand and make the cells to succumb to apoptosis under infection with ST-11 isolates. Sustained activation of JNK may not be the only reason for induction of apoptosis. Attenuation of NF-κB-regulated anti-apoptotic factors, such as cFLIP, that interacts and specifically inhibits the caspase-8, may also account for apoptosis promoted by ST-11 isolates.

Cleavage of p65/RelA occurs at the C-terminal region which contains the transactivation domain (TAD). Removal of the TAD from the rest of the protein may therefore disable the activation capacity of the native p65/RelA molecule. Alternatively, cleavage may generate a truncated N-terminal p65/RelA that can still bind DNA and thus potentially acts as a dominant-negative inhibitor by competing with intact p65/RelA. Bacterial proteins that target the nuclei of host cells may alter cell biology, which is an emerging pathogenic mechanism of bacteria [53,54]. Few bacterial proteins were found to induce host cell pathology through nuclear targeting including the cytolethal distending toxins CdtB of *Escherichia coli* and *Campylobacter jejuni* [55,56], the outer membrane protein OmpA and transposase of *Acinetobacter baumannii* [57,58], OspF of *Shigella* species [59] and recently two other meningococcal autotransporters App and MspA [60]. *Neisseria* IgA protease was previously reported to cleave human IgA1 [61], the lysosome-associated membrane protein 1 (Lamp1) promoting intracellular bacterial survival in epithelial cells *in vitro* [24,62,63], the human chorionic gonadotropin hormone (hCGH) and the membrane vesicular protein synaptobrevin II [25,64]. The biological significance of these processing remains unclear. Poliovirus protease 3C, the Chlamydial Protease-like Activity Factor (CPAF) and recently the type III secretion protease of enteropathogenic and enterohemorrhagic *Escherichia coli*, compromise NF-κB activation by targeting the cytosolic p65/RelA [65,66,67]. In contrast to these pathogens, the cleavage of p65/RelA by IgA protease of ST-11 meningococcal isolates occurs exclusively within the nuclear compartment. One possible explanation for this site specific effect could be the intracellular trafficking of this protease that may render it either temporarily inactive or inaccessible to p65/RelA within the cytosol. This issue relates to the mechanism of uptake and the intracellular trafficking of IgA protease. For instance, the IgA protease domain alone (IgaP) from non-ST-11 isolates was able to be internalized, as it could be detected intracellularly in the cytosolic fractions. These results suggest that this internalisation was mediated by the conserved protease domain, but cannot exclude a cooperative effective role of α peptide when associated to the protease domain.

Upon Nm-epithelial cells contact, the IgA protease is upregulated [68] allowing local production of substantial amounts of IgA protease, its accumulation in the nuclei of infected cells, the cleavage of p65/RelA and the induction of apoptosis. Nevertheless, absence of *iga* expression did not completely abolish the apoptotic effect promoted by ST-11 isolates. Other bacterial factors such as the outer membrane protein, PorB, that promotes apoptosis in epithelial cells by mitochondrial pathway [17,69] and autotransporter protein NhhA that has been demonstrated to trigger apoptosis in macrophages via an undefined mechanism [70] could be involved.

The α-peptide is a unique characteristic of pathogenic *Neisseriae* [29]. Our data and other reports revealed an extensive polymorphism within this region between ST-11 and non-ST-11 isolates [71,72,73]. Accordingly, José *et al*. described four different variants of meningococcal α proteins regarding the number of NLS and sequence variability. The majority of the strains analyzed in this report were isolated from nonsymptomatic carriers at different locations in Germany [73]. Our findings extended from previous investigations showed that the high diversity in some genetic lineages such as ST-32, correlated with heterogeneity in virulence for mice. In contrast, the homogeneous genetic structure of isolates of the clonal complex ST-11, regardless of their serogroup, correlated positively with a fatal outcome of the infection and increased virulence in mice [16], and pathogenic effects toward infected cells as determined by apoptotic assays [17]. Therefore, one can assume that apoptotic effect of some meningococcal strains but not others seems to be strain specific, which could be related in part to the capacity of some strains to protect their host cells from apoptosis. This polymorphism may explain the presence of two cleavage sites on IgA protease of ST-11 isolates and the production of two forms: an α-peptide-free smaller form, IgaP (~100 kDa) and a predominant α-peptide-linked peptidase longer form, IgaPα (~150-kDa). Non-ST-11 isolates release the only IgaP smaller form. Consistently, the *nalP* gene was in “phase on” status in all the isolates of our study, further suggesting that the absence of released IgaPα in non-ST-11 isolates is related to the lack of the cleavage site downstream α-peptide, leaving the α-peptide domain associated with the outer-membrane-embedded β core instead of being released extracellularly. A previous report suggested that NLS-carrying α-peptide of the gonococcal strain MS11 mediate nuclear targeting of reporter proteins but failed to detect IgA protease in the nuclear compartment of infected cells [29]. Indeed, MS11 gonococcal strain lacks a functional NalP protein that is responsible for release of IgA protease linked to α-peptide. Recently, Roussel-Jazédé *et al*. [74] reported the lack of NalP expression from the FAM18 strain, a ST-11 invasive isolate that harbor a second autocleavage site separating the α peptide from the translocator domain. FAM18 was unable to express a protease domain associated to α peptide (IgaPα). Consistently, the ratio of IgaPα to IgaP was strongly decreased in our NalP mutant of the ST-11 isolate LNP19995. Although NalP was not detected in the nuclear fractions of ST-11 infected cells (Fig 3C), our results may suggest a role of NalP in the down regulation of NF-κB activity most likely by modulating the alternative autocleavage site of IgA protease, providing therefore a stable passenger IgaPα able to interact with target cells and hence reach the nuclear compartment.

Our DsRed-chimeric fusion proteins and heterologous substitutions of *iga* alleles between ST-11 and non-ST-11 isolates further suggest that the attentive cleavage site by NalP is requested for NLS-harbouring α-peptide to act as carriers for nuclear transport of IgA protease to target the regulatory function of p65/RelA and probably other eukaryotic proteins.

Like many respiratory invasive bacterial pathogens, *N*. *meningitidis* colonize the epithelium of the nasopharynx, at the upper respiratory tract. Acquisition of *N*. *meningitidis* at this portal of entry may be asymptomatic or result in local inflammation [75]. A study after an epidemic in Burkina Faso reported that coughing, nasal congestion or sore throat during the preceding month were associated with carriage of the W135 associated outbreak strain that belong to the clonal complex ST-11 [76]. Induction of inflammatory response at this site could be beneficial for the invasive strains to induce cell death. Neutralization of infected cells by apoptosis may allow bacteria to persist or disseminate to deeper sterile sites. The modulation of the inflammatory response during meningococcal bacteraemia, may also enhance the death of endothelial cells and the permeability of the blood-brain barrier leading to dissemination of the bacteria to meninges [20]. Sustained NF-κB activity would allow commensal relationship of carriage isolates at their ecological niche through eradication of the alternative cleavage site on the IgA protease. This site present in the hyperinvasive ST-11 isolates may contribute to their ability to invade the host.

## Materials and Methods

### Reagents chemicals and antibodies

RPMI-Glutamax, Fetal calf serum (FCS), trypsin-EDTA and penicillin/streptomycin solution were purchased from Gibco Laboratories (Saint Aubin, France), FITC-Annexin V, and propidium iodide, were purchased from R & D Systems (Lille, France). Lipopolysaccharide of *E*. *coli* Serotype O55:B5, protease inhibitor cocktail tablets, phenylmethylsulfonyl fluoride (PMSF), protein A-agarose beads, cycloheximide and antibiotics were from Sigma (Saint-Quentin-Fallavier, France). Rabbit polyclonal to NF-κB p50, goat polyclonal antibody to C-terminal (residues 501–551), mouse monoclonal antibody to N-terminal (residues 136–224) NF-κB p65, rabbit polyclonal anti-histone H3 and anti-His6 tag mAb were obtained from abcam (Paris, France). Mouse anti-IκBα monoclonal antibody was purchased from Life technologies (Saint Aubin, France). Rabbit anti-GAPDH was from Sigma Aldrich (Saint-Quentin-Fallavier, France). Rabbit antibodies directed against JNK and its phosphorylated form were obtained from Cell signaling (Leiden, The Netherlands). HRP- and Texas red-conjugated secondary antibodies were purchased from Jackson immunoreseach (Marseille, France).

### Bacterial strains, growth conditions and preparation of meningococcal secreted proteins

*Escherichia coli* strain DH5α [77] was used for plasmid preparation and *E*. *coli* BL21(DE3) pLysS [78], was employed for overexpression of His_6_-tagged proteins. When indicated ampicillin, kanamycin, spectinomycin or chloramphenicol were added at final concentrations of 100 μg.ml^-1^, 50 μg.ml^-1^, 40 μg.ml^-1^ and 15 μg.ml^-1^, respectively. *N*. *meningitidis* clinical isolates in France are sent to the National Reference Centre for Meningococci (NRCM) for full typing. *N*. *meningitidis* was grown in GCB medium with Kellog supplements [79]. Phenotypes (serogroup: serotype: serosubtype) and MLST genotypes were determined as previously described [80]. Sequence types (ST) and clonal complexes were assigned using the *Neisseria* MLST database (http://pubmlst.org/neisseria). Phenotypic and genotypic characteristics of all *N*. *meningitidis* strains used in this study are listed in S1 Table.

Meningococcal Secreted proteins (MSPs) were prepared as previously described [81]. Preparations contained between 375–560 μg ml^−1^ proteins. Anti-PorA monoclonal antibodies (NIBSC, UK) were used to check for contaminating outer membrane proteins.

### Construction of IgA protease knockout, active site-directed mutagenesis, nalP knock out and complemented strains

Genomic and plasmid DNA were extracted using Qiamp DNA mini kit (Qiagen, Courtaboeuf, France) and PureLink Quick plasmid Miniprep Kit (Life Technologies, Saint Aubin, France), according to manufacturer instructions, respectively. All restriction and modification enzymes were used according to manufacturers’ recommendations. All primers used in this study were purchased from Sigma and are listed in S2 Table. As *iga* gene from LNP19995 and LNP21019 had not been sequenced, we selected primers based on conserved sequences in other meningococcal and gonococcal *iga* alleles described previously (strains MC58, Z2491 and Ng44/76 with accession numbers AE002424, AL162754 and X82481, respectively). The *iga* knockout construct was obtained as follows. Two DNA fragments of 534 bp and 475 bp (corresponding to positions 24 to 554 and 4950 to 5448 of MC58 *iga* orf, respectively) were amplified by PCR from chromosomal DNA of the stain LNP19995 using primer pairs iga5’Fw/iga5’Rev and iga3’FwEcoRI /iga3’RevNcoI, respectively. Both PCR products were purified, digested with appropriate restriction enzymes, when adapter sites were included in the primers, and sequentially cloned into pGEM-T-easy (Promega, Charbonnieres, France), with in between a PCR-generated spectinomycin-resistance cassette (*aadA*) from pHP45Ω-spec [82] that was inserted into the blunt-ended *EcoR*I site to yield plasmid pGEM-iga::aad. This plasmid was linearised by SalI restriction enzyme and transformed into LNP19995 (ST-11) and LNP21019 (non-ST-11) meningococcal isolates by homologous recombination and allelic replacement. Transformants were selected on 70 μg.ml^-1^ spectinomycin-supplemented GCB agar plates. The chromosomal configuration of spectinomycin-resistant strains was confirmed by PCR and DNA sequencing. One candidate from each transformation was named 19995*Δiga* and 21019*Δiga*, respectively and was selected for further analysis.

To generate complemented strains, *iga* gene was amplified using the primers iga5’Fw and iga3’RevNcoI (nucleotide 24 to 5448, according to MC58 nucleotide sequence) from LNP19995 or LNP21019 strains. The amplified fragments were inserted separately into pGEM-T-easy vector to generate the recombinant plasmids pGEM-iga_19995_ and pGEM-iga_21019_, respectively. Then, a 180 bp fragment corresponding to down-stream *iga* stop codon was generated by PCR using primers igadownFw-Nco and igadownRev and was inserted into blunt-ended *Nco*I site of pGEM-iga_19995_ or pGEM-iga_21019_ vectors. This insertion led to generate a new *Nco*I site in between *iga* and the fragment downstream. Finally, a blunt-ended erythromycin resistance cassette *erm* generated by PCR from the plasmid pMGC20 [83] using the primers ERAM1 and ERAM3, was inserted into the blunt-ended *Nco*I site. The leading recombinant plasmids were named pIga_19995_-erm and pIga_21019_-erm. These plasmids were linearised with SalI restriction enzyme and each plasmid was transformed into both strains 19995*Δiga* and 21019*Δiga*. Transformants that lost resistance to spectinomycin and acquired resistance to erythromycin were selected and named 19995*Δiga/iga*_*19995*_, 21019*Δiga/iga*_*19995*_ (for transformants generated with the plasmid pIga_19995_-erm) and 19995*Δiga/iga*_*21019*_ and 21019*Δiga/iga*_*21019*_, (for transformants generated with the plasmid pIga_21019_-erm), respectively. Correct replacement of deleted *iga* with the wild type alleles was verified by PCR and restored expression of IgA protease was confirmed by immunoblotting.

Inactivation by replacement of the active-site serine (^267^S) with a valine residue was accomplished by site-directed mutagenesis using the PCR-based megaprimer method [84]. The mutagenic primers igamutRev coding for replacement of serine with valine at position 267, was used in combination with the igapFwBsa primer (S2 Table). The 672 bp PCR product generated was gel purified and used as large forward primer (megaprimer) in a second PCR together with the reverse primer iga3’RevNcoI (S2 Table) to amplify the 5307-bp *iga* fragment. This product was subcloned into pGEM-T-easy to generate the plasmid pGEM-igaS^267^V. Then, the 180 bp fragment corresponding to down-stream *iga* stop codon was generated by PCR using primers igadownFw-Nco and igadownRev and was inserted into blunt-ended *Nco*I site. This insertion led to generate a new *Nco*I site in between mutated *iga* and the fragment downstream that was used to insert blunt-ended erythromycin resistance cassette leading to a recombinant plasmid pGEM-igaS267V. This plasmid was linearised with SalI restriction enzyme and was transformed into strain 19995*Δiga*. Transformants that lost resistance to spectinomycin and acquired resistance to erythromycin were selected and named 19995*iga* ^*S267V*^. Correct replacement of deleted *iga* with the S267V mutation was confirmed with PCR and DNA sequencing.

To generate a 19995Δ*nalP* mutant strains, 2175 bp *nalP* fragment extending from nucleiotides 473 to 2647 (according to MC58 orf) was amplified by PCR from LNP19995 genomic DNA using the primers nalPKOFw and nalPKORev (S2 Table). The PCR fragment was subcloned into pGEM-T-easy to generate the pasmid pGEM-nalP. The *erm* cassette conferring resistance to erythromycin was inserted into the blunt-ended restriction site of pGEM-nalP, resulting to the recombinant plasmid pGEM-nalP::erm. This vector was linearised with NcoI and was transformed into LNP19995. Transformants were then selected on plates containing erythromycin at 2 μg.ml^-1^. Correct inactivation of *nalP* gene was verified by PCR and immunoblotting using specific serum directed against NalP. One transformant was named 19995*ΔnalP* and was selected for further analysis.

### Construction of C-terminal His6- and DsRed-tagged IgA protease domains and purification of IgA protease domains

PCR-generated fragments encoding the protease domain (IgaP), α-peptide (Igaα) or both associated sub-domains IgaPα of IgA protease were generated from strains LNP19995 and LNP21019 using the couple of primers igapFwBsa/igapRevXho, alphaFwBsa/alphaRevXho and igapFwBsa/alphaRevXho, respectively. The PCR fragment encoding the protease domain harbouring the active site mutation S267V was also generated using the primers igapFwBsa/igapRevXho and pGEM-igaS267V as template. The PCR products were digested with BsaI and XhoI restriction enzymes and inserted between *Nco*I and *Xho*I restriction sites of the plasmid pET28b (Addgene, Middlesex, UK) to generate C-terminal His_6_-tagged fragments. The resulting recombinant plasmids (pIgaP_19995_, pIgaP_21019,_ pIgaα_19995_, pIgaα_21019_, pIgaPα_19995_ and pIgaPα_21019,_ respectively) were used to transform, over-express and purify the different cloned domains from *E*. *coli* BL21pLysS strain as described elsewhere [85]. To generate C-terminal DsRed-tagged domains, the same fragments were similarly amplified from each strain using the primers igapFwNhe/ igapRevSma, alphaFwNhe/ alphaRevSma and igapFwNhe/alphaRevSma. The resulting fragments were digested with NheI and SmaI restriction enzymes and inserted into pDsRedN1 (Clonetech, France) opened with the same enzymes to yield the recombinant plasmids pIgaP-Red_19995_, pIgaP-Red_21019,_ pIgaα-Red_19995_, pIgaα-Red_21019_, pIgaPα-Red_19995_ and pIgaPα-Red_21019_

### Preparation of mouse and rabbit sera

Anti-NalP antiserum was a generous gift from Dr. Isabel Delany (Novartis Vaccines, Research Center, Siena, Italy). To produce mouse serum against secreted Meningococcal proteins (MSPs), 6-week-old BALB/c mice were injected subcutaneously on days 1 with 30 μg of MSPs that were emulsified with Freund’s complete adjuvant (1:1, v/v) in a 500 μl total volume prior to injection. This injection was followed at days 14 and 21 by two MSPs booster injections in sterile PBS. On day 30, animals were test bled in comparison to pre-immune serum. Mice were sacrificed and sera were collected by cardiac puncture and stored at -20°C. Rabbit polyclonal serum against the protease sub-domain IgaP was prepared using purified His6-tagged IgaP of the strain LNP19995. Polyclonal serum was produced by immunizing a New Zealand White rabbit once with 30 μg of the purified protein mixed with Freund's complete adjuvant and twice with the same amount of antigen mixed with Freund's incomplete adjuvant at 2 week intervals. The animal was test bled 7 days after the third immunisation. The animal was sacrificed and the serum was stored at −20°C. Pre-immune serum was obtained before immunization. Before use in immunoblots, rabbit serum was pre-adsorbed for 3 h at 37°C with a 10% (v/v) suspension of each 19995*Δiga* and 21019*Δiga* mutants before being cleared by centrifugation at 14000 *g* at 4°C.

### Ethics statement

All experiments including immunizations and bleeding of animals were performed according to the European Union Directive 2010/63/EU (and its revision 86/609/EEC) on the protection of animals used for scientific purposes. Our laboratory has the administrative authorization for animal experimentation (Permit Number 75–1554) and the protocol was approved by the Institut Pasteur Review Board that is part of the Regional Committee of Ethics of Animal Experiments of the Paris region (CETEA 2013–0190).

### Cell culture, infection, transfections and luciferase reporter assay

Hec-1-B cells (American Type Culture Collection, Manassas, VA) were maintained in RPMI containing 10% FCS and 1X penicillin/Streptomycin until a confluence of 80%. A549 alveolar epithelial cells were cultured in DMEM (Invitrogen, France) plus 10% FBS, 2mM L-glutamine, 50 U/mL penicillin, and 1X penicillin/Streptomycin until 80–85% of confluence. Depending on experiments, cells were seeded in 24-well plates, 10-cm diameter dish plates or 75cm^2^ flasks at a density of 5.10^5^ cells/cm^2^ in antibiotic-free medium. Prior to infection, cells were washed and incubated with bacteria at bacteria to cell ratio of 25:1. When stated, infection was performed in presence of 20μg.ml^-1^ cycloheximide, 5 mM PMSF or 5 μg.ml^-1^ chloramphenicol (a concentration that was sufficient to block bacterial protein synthesis, as judged by growth inhibition, but low enough to allow survival of bacteria as judged by growth resumption, when chloramphenicol was removed).

All transfections were carried-out in OptiMEM medium (Life Technologies, France) in absence of antibiotics using Lipofectamine 2000 (Life Technologies, France) according to the manufacturer’s protocol. Plasmid p(Igκ)_3_conaluc was a generous gift from Pr. Alain Israel’s laboratory. Before transfection, cells were washed extensively and were then transfected with 2 μg/ml of each p(Igκ)_3_conaluc [86] and pCMVβ (Addgene, Middlesex, UK). Fourty eight hours post-transfection, cells were washed using PBS and then infected as indicated above. At indicated time points, luciferase activity was performed as described previously (*19*). Results are reported as relative luciferase units (RLU) after normalizing for β-galactosidase activity and protein concentration. For immunofluorescence microscopy, cells were first grown on 12-mm glass coverslips. Adherent cells were then transfected with 2 μg/ml of DsRed fusion constructs as before and then proceeded for immunofluorescence microscopy.

### Extraction of RNA, cDNA synthesis, and quantitative real-time-PCR (qRT-PCR)

Total RNA was extracted from infected or uninfected Hec-1-B monolayer cells at indicated time points using RNeasy Mini Kit (50) (Promega, France) according to the manufacturer's instructions. Two hundred nanograms of RNA were used as the template in the reverse transcription reaction. The total cDNA was obtained by reverse transcription PCR using the oligo(dT)_18_ primer and AMV reverse transcriptase (Biolabs, France) as described elsewhere [87]. The mRNA sequences for the target genes (IL-8, TNF-*α*, and cFLIP and *β*-actin of *Homo sapiens*) were obtained from the GenBank database (http://www.ncbi.nlm.nih.gov/). *β*-Actin was used as an internal control. Specific primers were designed using Primer Premier 5.0 (S2 Table) and synthesized by Sigma Aldrich (France). The qPCR reaction was performed on an Applied Biosystems model StepOne plus in triplicate using 25 ng of cDNA and SYBR green Universal PCR Master Mix in a total volume of 25 μl. The program consisted of an initial denaturation at 95°C for 10 min, followed by 40 cycles of 95°C for 15 s and 60°C for 1 min. Quantification of the targets was performed relative to uninfected sample using β-actin as internal control and the 2^−ΔΔCT^ method [88].

### Flow cytometry sorting of intact nuclei

Intact nuclei were sorted by adapting a previously described flow cytometer sorting-based technique [89]. Briefly, Hec-1-B cells infected with GFP-expressing bacteria (S1 Table) or left uninfected were washed carefully with PBS and harvested by centrifugation at 1500 *g* for 10 min. Unless otherwise indicated, the following steps were carried-out at 4°C. Cells were resuspended in 1 ml phosphate-buffered salin (PBS), pH 7.5; supplemented with a cocktail of protease inhibitors and 50 μg.ml^-1^ RNase A (Thermo Scientific, France). Cells were homogenized by several strokes through a 26 gauge-needle attached to a 1 ml syringe until 80–90% lysis was reached as judged under light microscope. Samples were then centrifuged for 15 min at 1500 rpm. Supernatant containing cytosolic fraction was saved at -80°C until use and the pellet containing a mix of free nuclei, cell debris, unbroken cells and bacteria, was carefully resuspended in 500 μl PBS and stained for 15 min with 5 μg.ml^-1^ propidium iodide (PI, red fluorescence) at 4°C in the dark to label free nuclei or cells that lost membrane integrity. After staining, samples were washed with PBS, pelleted for 15 min at 1500 rpm and resuspended in 1 ml PBS. Samples were analyzed using a FACSCalibur flow cytometer (BD Biosciences) equipped with an argon laser emitting at 488_nm_ for the excitation of PI and GFP. Preliminary control experiments included non-labelled bacteria, GFP-expressing bacteria, lysed or intact cells stained or not with PI, confirming location of the fractions in the homogenized sample. Free nuclei were first discriminated from intact cells by SS (side scatter), FS (forward scatter) and FL2 (red fluorescence) parameters. GFP-positive (FL-1) events characterizing bacteria were then excluded from the sorted gate. Then, free nuclei defined as PI-positive and GFP–negative populations were gated in the region R4 (See S3 Fig) and were sorted using single cell sorting mode adjusted to 250–300 events/s, thereby limiting the generation of coincidental events. Sorted population was re-analyzed to confirm purity. Relative intensities of SS, FL1 and FL2 were recorded as log scale, 1024 channels and 4 decades. Relative intensities of FS was recorded as linear scale. To validate the sorted population, nuclei were examined by immunofluorescence microscopy after DAPI staining and immunoblotted for specific nuclear markers.

### Two dimensional gel electrophoresis

Sorted nuclei were pelleted at 3000 rpm for 15 min at 4°C, resuspended in 150 μl rehydration buffer (8 M urea, 2% CHAPS, 2% IPG buffer, 20 mM DTT and 0.002% Bromophenol Blue) and analyzed by two dimensional gel electrophoresis (2DGE) as described elsewhere [90]. Proteins were first separated according to their isoelectric point (pI) in the immobilin Dry-Strip pH 3–10 (Biorad) using IPGphor isoelectric focusing system (Amersham Biosciences, Piscataway, NJ). After equilibration, proteins separated on the strips were layered on 12% SDS-PAGE gels and subjected to electrophoresis. Gels were then subjected to immunoblotting using mouse serum directed against secreted meningococcal proteins, rabbit serum specific to IgA protease domain or respective pre-immune sera.

### In vitro p65/RelA and IgA protease cleavage assays and IgA protease activity

Hec-1B cells were stimulated for 1h with 10 μg.ml^-1^ LPS of E. coli 0111:B4. Nuclear extracts were prepared as described by Philpott et al. [91] and were used as a source of translocated p65/RelA. One microgram of this fraction was incubated with increasing amounts of purified IgA protease domains IgaP, Igaα, or IgaPα in a final volume of 20 μl of reaction buffer (10 mM Tris-HCl pH 7.4, 150 mM NaCl, 0.5 mM DTT, 2.5 mM CaCl_2_, and 0.5 mM MgCl_2_). In another set of experiments, 500 ng of purified IgaPα_19995_ or IgaPα_21019_ were incubated with 5 or 10 μg of MSPs from LNP19995 or LNP21019, respectively in a final volume of 20 μl of the same reaction buffer. The reaction mixtures were incubated at 25°C for 3 h. The reaction was stopped with 1 X Laemmeli buffer and separated in 10% SDS-PAGE before immunobloting using appropriate antibody (anti-p65/RelA antibodies or anti-His tag mAb). IgA protease activity was performed as described elsewhere [92].

### Apoptosis assays

Adherent cells were washed carefully, harvested by centrifugation at 1500 *g* for 5 min at 4°C and stained with 10 μl FITC-conjugated annexin V and 5 μg/ml propidium iodide (PI) and analyzed using flow cytometer FACSCalibur (BD Biosciences, Germany) as indicated elsewhere (*18*). Data were analyzed using Cyflogic software v.1.2.1.

### Immunofluorescence microscopy

Cells were fixed for 20 min in 3.7% paraformaldehyde in PBS for 15 min. When indicated, cells were permeabilised with 0.5% Triton-X in PBS containing 2% BSA, and subsequently stained for immunofluorescence microscopy as described before (*18*). Slides were then viewed with a conventional immunofluorescence microscope Zeiss Axio Imager D1 coupled to AxioCam MRm vers.3 (Carl Zeiss, Germany). Digital images were acquired using appropriate filters and combined using the Axiovision Rel. 4.6 software (Carl Zeiss).

### Co-immunoprecipitation, cell fractionation and immunoblot analysis

Cell fractions (cytosolic and nuclear fractions) were prepared as described elsewhere [91]. LNP19995 and *iga* deletion mutant 19995*Δiga* were grown in GC medium base until midlog phase. Then bacteria were centrifuged and the supernatant was concentrated 50 X nd incubated with 500 μg of nuclear extracts prepared from LPS-stimulated Hec-1-B cells (that served a source of translocated NF-κB) in presence of 5 mM PMSF as serine protease inhibitor. After 6h of incubation, 5 μg of anti-IgaP rabbit serum (or rabbit irrelevant antibody) or 1 μg of anti-N-terminal p65/RelA mAb (or irrelevant mouse IgG mAb antibody) were added and immunoprecipitation was performed as described elsewhere [93]. Protein complexes were solubilised in 1× Laemmeli buffer, resolved by SDS-PAGE and transferred to polyvinylidene difluoride (PVDF) membrane that was probed with appropriate primary antibodies. The immunoreactive band was visualized using appropriate HRP-conjugated secondary IgG antibody and ECL detection reagents (Amersham Pharmacia Biotech, France). The membranes were visualized using *ChemiDoc XRS* imager and QuantityOne 4.6 software (Bio-Rad).

### Total RNA preparation and reverse-transcription-polymerase chain reaction (RT-PCR)

Total RNA was isolated from plate-grown bacteria using a hot-phenol method described by Ducey *et al*. [94]. cDNA specific to *trpB* gene (encoding tryptophane synthase beta chain) downstream *iga* gene was generated from 2 μg total RNA with trpBRev primer using SuperScript II RT-PCR kit according to manufacturer instructions (Invitrogen). The PCR step was performed using trpBFw and trpBRev primers (S2 Table). Expression of *porA* was performed as internal control using primers porA0 and porA101 (S2 Table).

### DNA sequencing and sequence analysis

The genome databases of strain MC58 was interrogated using the server available at http://microbes.ucsc.edu/lists/neisMeni_MC58_1/refSeq-list.html. PCR products were sequenced by Eurofins-Cochin (Paris, France). DNA and protein sequence analysis was carried out using BioEdit Sequence Alignment Editor Software Version 7.2.5 available at http://www.mbio.ncsu.edu/bioedit/bioedit.html.

### Statistical analysis

Data were analyzed by two-way ANOVA test using the software GraphPad Prism version 4.00 for Windows (Graph-Pad Software, San Diego, California, USA, www.graphpad.com). *P* < 0.05 was considered statistically significant.

## Supporting Information

S1 Table *N*. *meningitidis* strains used in this study. (PDF)

S2 Table Primers used in this study. (PDF)

S1 Fig Analysis of IκB and NF-κB expression. Hec-1-B epithelial cells were left uninfected or infected with a subset of pathogenic ST-11 or non-ST-11 isolates. After 1 h or 9 h of incubation, cells were harvested and (A) IκBα expression was examined by immunoblotting from cytosolic fractions. GAPDH was used as loading control. (B) Nuclear fractions were probed with anti-N terminal or anti-C-terminal of p65 specific antibodies. Histone H3 was used as loading control. (C) Nuclear fractions of A549 cells were prepared after 9 h of infection with the indicated ST-11 or non-ST-11 isolates and examined by immunoblotting using anti-N terminal of p65 specific antibody.
(TIF)

S2 Fig Secreted meningococcal proteins may reach the nuclear compartment of infected cells. (A) Schematic description of gating strategy adopted to sort intact nuclei. Hec-1-B cells were infected with GFP-expressing LNP19995, or left uninfected. After infection, cells were prepared by several strokes through 26-gauge needle syringe and stained with propidium iodide as described in Materials and Methods. In *a* and *a’*, cell population was localized by plotting forward scatter channel (FSC) on a linear scale versus side scatter channel (SSC) on a log scale. Dot plots indicate two distinct populations that were sharply distinguishable based on their configuration. Intact cells fall into region R1 and cell debris in region R2 (*a* and *a’*). When homogenised, the massive population fall into region R2 (*b* and *b’*). Bacteria alone were also localized in region R2 using similar SSC and FSC parameter settings (*b”*). Nevertheless, bacteria could be easily distinguished by green fluorescence (FL1) (*c”*, region R3). In *c* and *c’*, the fluorescence of population gated in region R2 was analysed after PI staining on channels FL1 (GFP) and FL2 (PI). In that gated population, free nuclei (PI^+^/GFP^-^) were readily discriminated in region R4 from bacteria (GFP^+^) in region R3 and cell debris (PI^-^/GFP^-^). The population gated in region R4 was therefore sorted and purity was re-examined after sorting (*d* and *d’*). The sorted nuclei were confirmed cytologically using DAPI staining and immunofluorescence microscopy examination (e and *e’*). In *f* the quality of sorted nuclei was assessed by immunoblotting using specific cytosolic and nuclear markers GAPDH and histone H3, respectively. N, sorted nuclei; C, cytosolic fraction. (B) Immunoblot analysis of a 2D-gel electrophoresis of flow cytometry-sorted nuclei. Proteins of sorted nuclei from uninfected (+ RPMI) or LNP19995-infected (12 h; MOI: 25) Hec-1-B cells (+LNP19995), were fractionated over immobilized pH gradient from (pH 3 to 10) in the horizontal dimension, followed by fractionation by SDS-PAGE in the vertical dimension. Gels were then transferred onto nitrocellulose membrane and were immunoblotted using mouse serum anti-MSP (*a* and *c*, upper panel) or rabbit anti-IgA protease sub-domain (*b* and *d*, upper panel), respectively. Mouse and rabbit pre-immune sera (lower panel) were used as negative controls.
(TIF)

S3 Fig Nuclear cleavage of p65 correlated with the nuclear expression of IgA protease of ST-11 but not non-ST-11 isolates. (A) Hec-1-B cells were infected with a subset of ST-11 or non-ST-11 isolates. After 12 h cytosolic fractions were prepared. In parallel, MSP of the indicated isolates were prepared as indicated in Materials and Methods. Samples were resolved by SDS-PAGE and then examined by immunoblot using the polyclonal serum to IgaP or NalP. Positions of both IgA protease secreted forms are indicated by arrows. The molecular weight is presented. (B) The nuclear fractions of infected cells from (A) were prepared, resolved by SDS-PAGE and then immunoblotted with anti-p65 mAb (N-terminal specific) or the polyclonal serum to IgaP.
(TIF)

S4 Fig Characterization of the WT, *iga* knock-out mutants and complemented strains. (A) MSPs were prepared from the indicated strains and resolved in SDS-PAGE. After transfer, the membrane was probed against anti-IgaP specific serum. Both IgA protease secreted forms are indicated with arrows. (B) Insertion of spectinomycin resistance cassette in *iga* has no polar effect on expression of *trpB* downstream *iga*. RT-PCR analysis was performed from total RNA extracted from the indicated strains. Samples were analysed on 2.5% agarose gel and stained with ethidium bromide before visualisation by UV transluminator. Expression of *porA* was used as an internal control. (C) Alteration of NF-κB transcriptional activity is compromised in epithelial cells infected with *iga*-knock-out mutant of ST-11 isolates. Hec-1-B cells were co-transfected with p(Igκ)_3_conaluc and pCMVβ prior to infection. After 48 h, cells were infected with the indicated strains. After each time point, cells were harvested and luciferase activities were determined. Data (mean ± SD) are presented as relative luciferase units (RLU). ***, *P* < 0.001 for a comparison of cells infected with the wild type ST-11 isolate or isogenic complemented strains and those infected with the *iga* mutant or LNP21019 carriage isolate. (D) Whole bacterial lysates and secreted proteins were prepared from the indicated strains, then resolved in SDS-PAGE and examined by immunobloting using the serum directed against the IgA protease domain. The full length precursor and the cleavage products are indicated by arrows. The molecular sizes of each form is indicated. * represents a cross-reactive band. (E) Left panel: DNA sequence chromatograms of the wild type LNP19995 (WT) and the 19995*iga* ^*S267V*^ mutant showing the AG to GT substitution (in square). Right panel: Expression of LNP19995 IgA protease WT and mutants 19995*Δiga* and 19995*iga* ^*S267V*^. Enzyme activity was measured by quantitative ELISA with human IgA1 as a substrate, as described in Materials and Methods. Sterile medium as negative control showed no activity. The error bars indicate mean ± SD from three independent experiments.
(TIF)

S5 Fig Over-expression and purification of IgA protease passenger sub-domains. The whole passenger domain IgaPα or each sub-domain IgaP and Igaα (A) and IgaP^S267V^ (B) were subcloned into the vector pET28b from each strain LNP19995 and LNP21019 and were over-expressed and purified from BL21 (DE3) pLyS as C-terminal His6-tagged proteins after induction with 1mM IPTG at 37°C for 2 hours. The total cell lysates and purified proteins were analyzed with SDS-PAGE and blue Coomassie brilliant stain (left panel). Over-expressed proteins are indicated with blue arrowheads. Purified proteins were also confirmed by immunoblotting using anti-His tag specific mAb. The molecular weight (kDa) is shown in the left side.
(TIF)

S6 Fig Comparison of α-peptide amino acid sequences between pathogenic ST-11 and Non-ST-11 meningococcal isolates. The amino acid sequences were deduced from the α-peptide encoding regions of the strains indicated in the left. The alignment was performed with BioEdit program. Gaps, indicated by dashed lines, were introduced by the program. Identical amino acids are in black letters on a yellow background and asterisks in the bottom of alignments. Similar residues are indicated in black letters on grey background. Auto-proteolytic cleavage sequences CS1 and CS2 upstream and downstream α-peptide, respectively, are indicated in black letters on red background. Cleavage sites are indicated by arrowheads. Bipartite NLS sequences are indicated with blue arrows above the sequences. Upstream and downstream sequences of each NLS cluster are indicated in white letters on blue background. The amino acid positions are indicated according to the top sequence. The start of α peptide, the linker and β core (Igaβ) subdomains are indicated.
(PDF)
