# Supplementary material for: Hyperinvasive Meningococci Induce Intra-nuclear Cleavage of the NF-κB Protein p65/RelA by Meningococcal IgA Protease
Source: PLoS Pathog. 2015 Aug 4;11(8):e1005078. doi: 10.1371/journal.ppat.1005078 (PMC4524725; doi:10.1371/journal.ppat.1005078)
Supplement: S1 Table — (PDF) [file ppat.1005078.s001.pdf]

Table S1 *N. meningitidis* strains used in this study.

Strain	Characteristics: Serogroup :Serotype :Serosubtype (cc[1])	Isolation site	Origin
**ST-11 isolates**			
LNP13143	W: 2a: P1.5,2 (cc:11)	CSF[2]	NRCM[3]
LNP 17592	W: 2a: P1.5,2 (cc:11)	Blood	NRCM
LNP19008	C: 2a: P1.5, 2 (cc:11)	Blood	NRCM
LNP 19995	W: 2a:P1.5, 2 (cc:11)	CSF	NRCM
LNP 20342	B: 2a: P1. 5 (cc:11)	CSF	NRCM
LNP 20553	C: 2a: P1.5 (cc:11)	CSF	NRCM
LNP21515	C:2a: P1. 5 (cc:11)	CSF	NRCM
LNP21678	C:2a: P1. 5 (cc:11)	Blood	NRCM
LNP21996	B: 2a: P1.5 (cc:11)	Blood	NRCM
LNP24198	C:2a:P1.7, 1 (cc:11)	Blood	NRCM
LNP25496	C: 2a: P1.5 (cc:11)	CSF	NRCM
LNP25501	C:2a:P1.5 (cc:11)	CSF	NRCM
LNP25512	C:NT[4]: NST[5] (cc:11)	Blood	NRCM
LNP25516	C: 2a: P1.5 (cc:11)	Blood	NRCM
LNP25524	C:2a: P1. 5 (cc:11)	Blood	NRCM
LNP26653	C:15: P1.4 (cc:11)	Blood	NRCM
LNP27531	W:14: P1. 5, 2 (cc:11)	Synovial fluid	NRCM
LNP27557	B: NT: P1. 7, 1 (cc:11)	Blood	NRCM
LNP27564	W: 2a: P1.5, 2 (cc:11)	Blood	NRCM
**Carriage isolates**			
LNP117	NG[6]:NT:P1.6 (cc:22)	Nasopharynx	
LNP124	NG: 15: P1.6 (cc:NA[7])	Nasopharynx	NRCM
LNP128	NG: 1: P1.6 (cc:41/44)	Nasopharynx	NRCM
LNP133	29E: NT: P1.5, 2 (cc: 60)	Nasopharynx	NRCM
LNP280	B: NT: P1.9 (cc: 41/44)	Nasopharynx	NRCM
LNP292	Y: 15: P1.16 (cc:174)	Nasopharynx	NRCM
LNP597	NG: 1: P1.6 (cc: 41/44)	Nasopharynx	NRCM
LNP858	B: 1: P1.6 (cc: 461)	Nasopharynx	NRCM

LNP1128	B: 1: P1.14 (cc: 213)	Nasopharynx	NRCM
LNP1185	NG: NT:NST: (cc:NA)	Nasopharynx	NRCM
LNP1407	B: NT: P1.14 (cc: 213)	Nasopharynx	NRCM
LNP1632	B: 15: P1.7, 16 (cc: 32)	Nasopharynx	NRCM
LNP1769	NG: 15: P1.6 (cc: NA)	Nasopharynx	NRCM
LNP2073	B: 4: P1.14 (cc: 162)	Nasopharynx	NRCM
LNP2404	B: NT: NST (cc: 41/44)	Nasopharynx	NRCM
LNP3174	NG: 15: P1.6 (cc: NA)	Nasopharynx	NRCM
LNP 20642	C: NT: NST (cc: 334)	Expectoration	NRCM
LNP 21019	B: NT:P1.14 (cc: 35)	Expectoration	NRCM
**Recombinant meningococcal strains**			
1999Δiga	Isogenic mutant of the invasive isolate LNP19995, deficient in IgA protease expression (*iga::aad*), Spec^R [8]	N/A	This study
21019Δiga	Isogenic mutant of the carriage isolate LNP21019, deficient in IgA protease expression (*iga::aad*), Spec^R	N/A	This study
19995$\Delta iga/iga_{19995}$	Recombinant strain that resulted from substitution of chromosomal *iga::aad* copy of 19995Δiga with intact *iga* allele of LNP19995, Ery^R [9]	N/A	This study
19995$\Delta iga/iga_{21019}$	Recombinant strain that resulted from substitution of chromosomal *iga::aad* copy of 19995Δiga with intact *iga* allele of LNP21019, Ery^R	N/A	This study
21019$\Delta iga/iga_{19995}$	Recombinant strain that resulted from substitution of chromosomal *iga::aad* copy of 21019Δiga with intact *iga* allele of LNP19995, Ery^R	N/A	This study
21019$\Delta iga/iga_{21019}$	Recombinant strain that resulted from substitution of chromosomal *iga::aad* copy of 21019Δiga with intact *iga* allele of LNP21019, Ery^R	N/A	This study
19995GFP	Recombinant strain isogenic of LNP19995, expressing constitutively a chromosomal copy of green fluorescent protein GFP	N/A	(*18*)

1 : Clonal complex, 2 : Cerebrospinal fluid, 3 : National Reference Centre for Meningococci, 4 : Non-typeable, 5 : Non-serosubtypeable, 6 :Non-groupeable, 7 : Non assigned, 8 : Spectinomycin resistant, 9: Erythromycin resistant.
