# Supplementary material for: Hyperinvasive Meningococci Induce Intra-nuclear Cleavage of the NF-κB Protein p65/RelA by Meningococcal IgA Protease
Source: PLoS Pathog. 2015 Aug 4;11(8):e1005078. doi: 10.1371/journal.ppat.1005078 (PMC4524725; doi:10.1371/journal.ppat.1005078)
Supplement: S2 Table — (PDF) [file ppat.1005078.s002.pdf]

Table S2 Primers used in this study.

**Primer**	**Sequence**	**Features**
iga5'Fw	5'-TAACGCCATATCCTTATCC-3'	Coding strand of *iga5'* fragment (corresponding to position 24 to 48 of MC58 *iga* orf)
iga5'Rev	5'-GAAAAACGGTTTTTATCTTT	Non-coding strand of *iga5'* fragment (corresponding to position 538 to 557 of MC58 *iga* orf)
iga3'FwEcoRI	5'-ATCGAATTCCGGCGATATTGGTGCAGGCAGCTTG-3'	Coding strand of *iga3'* fragment (corresponding to position 4188 to 4212 of MC58 *iga* orf) harbouring *Eco*RI adaptor site (underlined sequence)
iga3'RevNcoI	5'-CCCCCATGGTTAGAAACGAATCTGTATTT-3'	Non-coding strand of *iga3'* fragment (corresponding to position 4662 to 4686 of MC58 *iga* orf) harbouring *Nco*I adaptor site (underlined sequence)
aad1	5'-TGCGCTCACGCAACTGGTCCA-3'	Coding strand of spectimomycin resistance cassette *aadA*
aad2	5'AGCTT*GCCGTCTGAA*GAATTCCCGGGGATCCGGTG-3'	Non-coding strand of spectimomycin resistance cassette *aadA* with up-take sequence (*italic*)
ERAM-1	5'-GCAAACTTAAGAGTGTGTTGA-3'	Coding strand of erythromycin resistance cassette *erm*
ERAM-3	5'-AGCTT*GCCGTCTGAA*TGGGACCTCTTTAGCTTCT-3'	Non-coding strand of erythromycin resistance cassette *erm*, with up-take sequence (*italic*)
alphaFwBsa	5'- GCTGGTCTCCCATGAGCCCGCAGGCAAATCA-3'	Coding strand of α-peptide region harbouring *Bsa*I adaptor site (underlined sequence)
alphaRevXho	5'- CACCTCGAGGGTATTGGTGCTGACTGCATC-3'	Non-coding strand of α-peptide region harbouring *Xho*I adaptor site (underlined sequence)
igapFwBsa	5'-GCTGGTCTCCCATGGCATTGGTCAGAGACGATG-3'	Coding strand of IgA protease sub-domain harbouring *Bsa*I adaptor site (underlined sequence)

igapRevXho	5'-CACCTCGAGAGGCGGTGCGACGACGATATT-3'	Non-coding strand of IgA protease sub-domain harbouring *Xho*I adaptor site (underlined sequence)
alphaFwNhe	5'- GCTGCTAGCATGAGCCCGCAGGCAAATCA-3'	Coding strand of α-peptide region harbouring *Nhe*I adaptor site (underlined sequence)
alphaRevSma	5'-CACCCCGGGGGTATTGGTGCTGACTGCATC-3'	Non-coding strand of α-peptide region harbouring *Sma*I adaptor site (underlined sequence)
igapFwNhe	5'-GCTGCTAGCATGGCATTGGTCAGAGACGATG-3'	Coding strand of IgA protease sub-domain harbouring *Nhe*I adaptor site (underlined sequence)
igapRevSma	5'-CACCCCGGGAGGCGGTGCGACGACGATATT-3'	Non-coding strand of IgA protease sub-domain harbouring *Sma*I adaptor site (underlined sequence)
trpBFw	5'-GGGTAAAAAACGCGTCATCG-3'	Coding strand of meningococcal *trpB* gene
trpBRev	5'-GCGATTTCCAATGCGTGATT-3'	Non-coding strand of meningococcal *trpB* gene
porA0	5′-GATGTCAGCCTATACGGCGAAATCAAA-3′	5′ of the coding strand of *porA*
porA101	5′-GCCGATAAACGAGCCGAAATC-3′	3′ of the non-coding strand of *porA*
igadownFw-Nco	5'-GGGCCCCCATGGAATACTAAATTCATAGCAAA-3'	Coding strand downstream *iga* stop codon
igadownRev	5'-CCTCGTCCGTCTGATATAGT-3'	Non-coding strand downstream *iga* stop codon
nalPKOFw	5'-ACGGCTATAACGAAAATTAC-3'	Coding strand *nalP*
nalPKORev	5'-TAATGCTGTCGGTTTTTGC-3'	Non-coding strand *nalP*
igamutRev	5'-TAATGGTGAACCGACATCGCCTAACA-3'	Non-coding strand *of iga with CT to AC replacement of active site (underlined)*
β-actinFw	5'- GACTTAGTTGCGTTACACCCTTTC-3'	β-actin coding strand (Accession number NM_001101.3)
β-actinRev	5'- CTGCTGTCACCTTCACCGTTC-3'	β-actin non-coding strand
TNF-αFw	5'- AGCTGGAGAAGGGTGACCGA-3'	TNF-α coding strand (Accession number: NM_000594)
TNF-αRev	5'- CAGGGCAATGATCCCAAAGTA-3'	TNF-α non-coding strand
IL-8Fw	5'- AGACATACTCCAAACCTTTCCACC-3'	IL-8 coding strand (Accession number NM_000584)

IL-8Rev	5'ACAACCCTCTGCACCCAGTT-3'	IL-8 non-coding strand
FLIPFw	5'-AGAGTGAGGCGATTTGACCTG-3	c-FLIP coding strand (Accession number HS00094)
FLIPRev	5'- -AAGGTGAGGGTTCCTGAGCA-3'	c-FLIP non-coding strand
