# Supplementary figures and images for: Hyperinvasive Meningococci Induce Intra-nuclear Cleavage of the NF-κB Protein p65/RelA by Meningococcal IgA Protease
Source: PLoS Pathog. 2015 Aug 4;11(8):e1005078. doi: 10.1371/journal.ppat.1005078 (PMC4524725; doi:10.1371/journal.ppat.1005078)

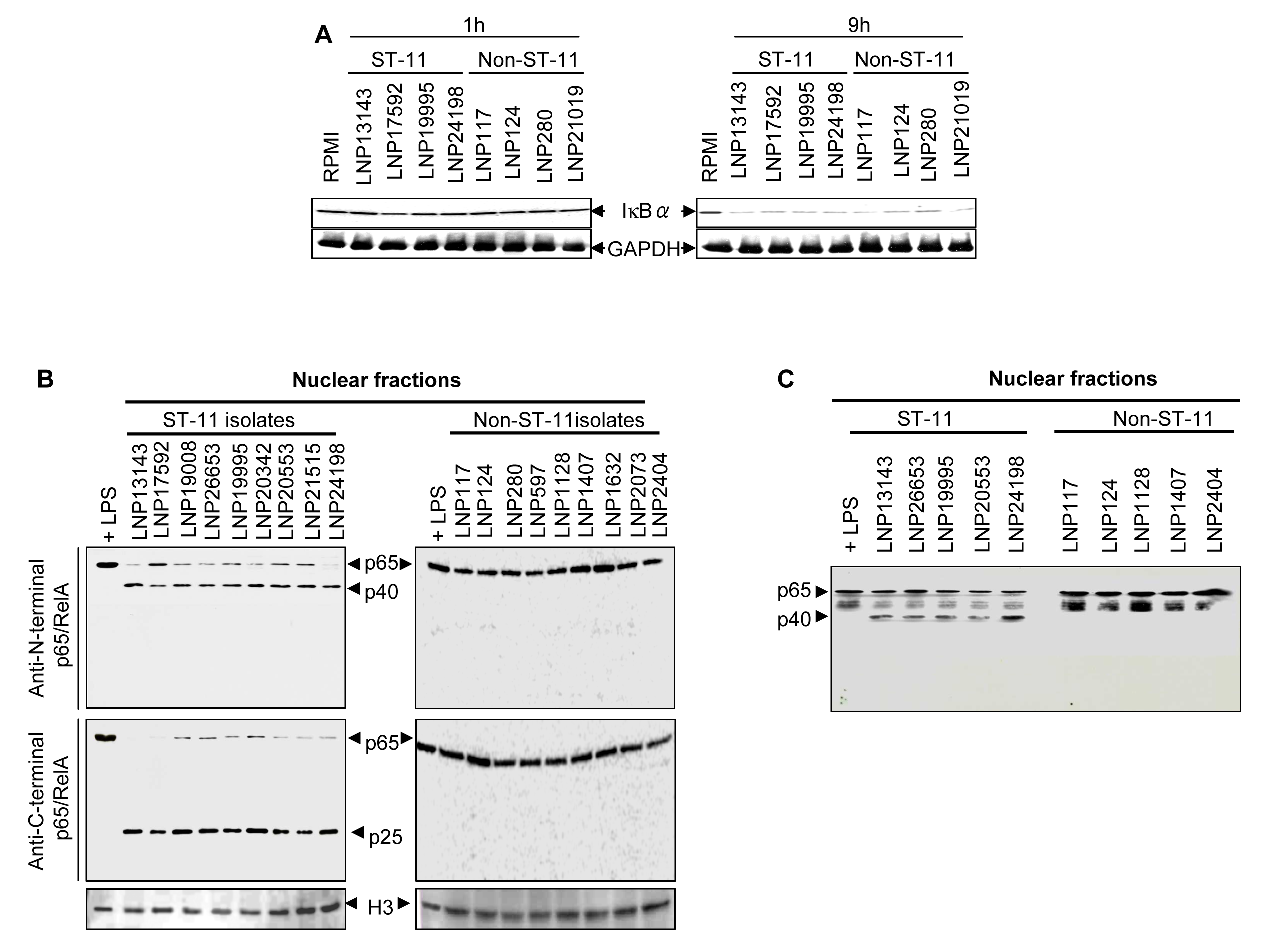

Supplement: S1 Fig — Hec-1-B epithelial cells were left uninfected or infected with a subset of pathogenic ST-11 or non-ST-11 isolates. After 1 h or 9 h of incubation, cells were harvested and (A) IκBα expression was examined by immunoblotting from cytosolic fractions. GAPDH was used as loading control. (B) Nuclear fractions were probed with anti-N terminal or anti-C-terminal of p65 specific antibodies. Histone H3 was used as loading control. (C) Nuclear fractions of A549 cells were prepared after 9 h of infection with the indicated ST-11 or non-ST-11 isolates and examined by immunoblotting using anti-N terminal of p65 specific antibody. (TIF) [file ppat.1005078.s003.tif]

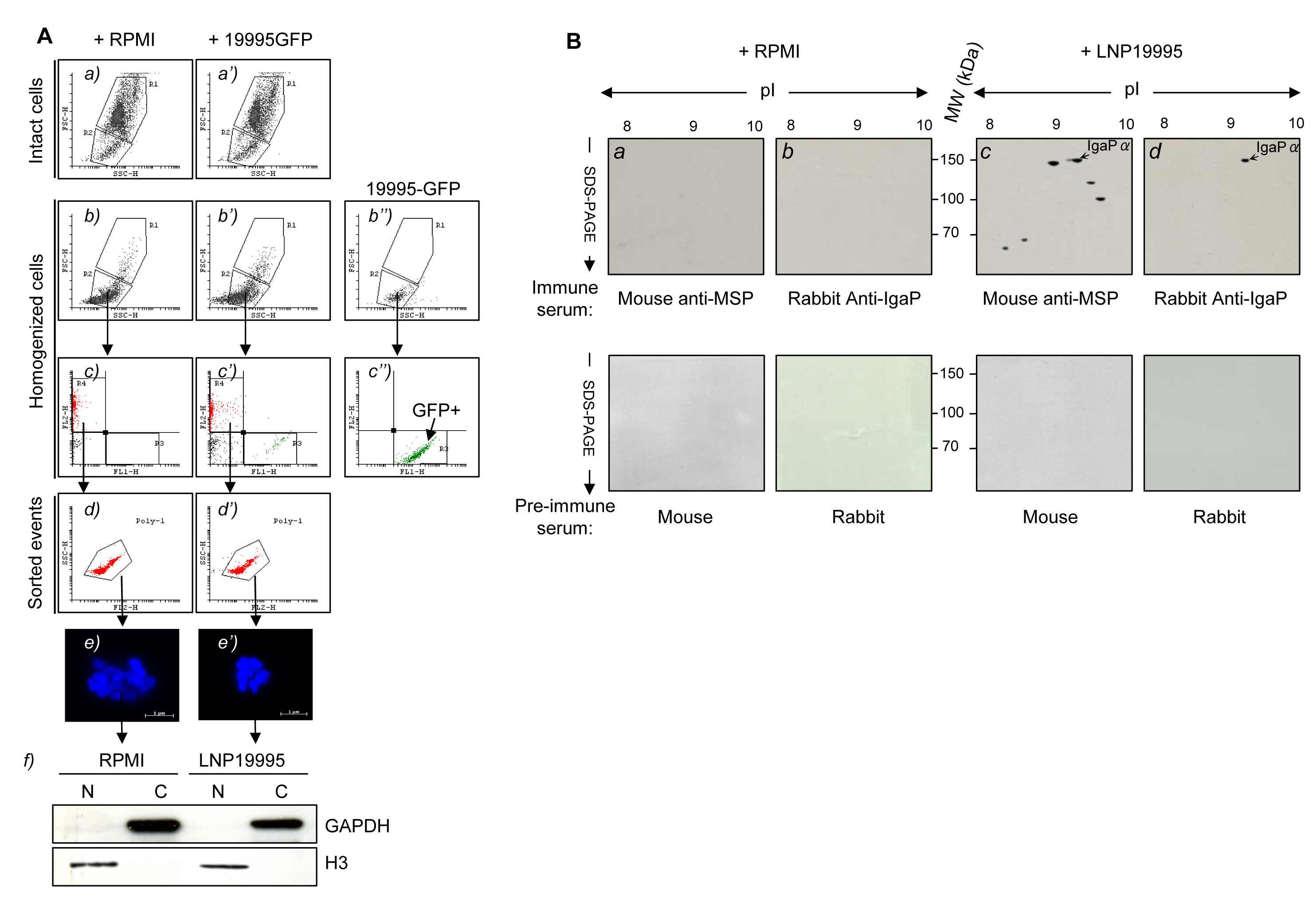

Supplement: S2 Fig — (A) Schematic description of gating strategy adopted to sort intact nuclei. Hec-1-B cells were infected with GFP-expressing LNP19995, or left uninfected. After infection, cells were prepared by several strokes through 26-gauge needle syringe and stained with propidium iodide as described in Materials and Methods. In a and a’, cell population was localized by plotting forward scatter channel (FSC) on a linear scale versus side scatter channel (SSC) on a log scale. Dot plots indicate two distinct populations that were sharply distinguishable based on their configuration. Intact cells fall into region R1 and cell debris in region R2 (a and a’). When homogenised, the massive population fall into region R2 (b and b’). Bacteria alone were also localized in region R2 using similar SSC and FSC parameter settings (b”). Nevertheless, bacteria could be easily distinguished by green fluorescence (FL1) (c”, region R3). In c and c’, the fluorescence of population gated in region R2 was analysed after PI staining on channels FL1 (GFP) and FL2 (PI). In that gated population, free nuclei (PI+/GFP-) were readily discriminated in region R4 from bacteria (GFP+) in region R3 and cell debris (PI-/GFP-). The population gated in region R4 was therefore sorted and purity was re-examined after sorting (d and d’). The sorted nuclei were confirmed cytologically using DAPI staining and immunofluorescence microscopy examination (e and e’). In f the quality of sorted nuclei was assessed by immunoblotting using specific cytosolic and nuclear markers GAPDH and histone H3, respectively. N, sorted nuclei; C, cytosolic fraction. (B) Immunoblot analysis of a 2D-gel electrophoresis of flow cytometry-sorted nuclei. Proteins of sorted nuclei from uninfected (+ RPMI) or LNP19995-infected (12 h; MOI: 25) Hec-1-B cells (+LNP19995), were fractionated over immobilized pH gradient from (pH 3 to 10) in the horizontal dimension, followed by fractionation by SDS-PAGE in the vertical dimension. Gels were then trans [file ppat.1005078.s004.tif]

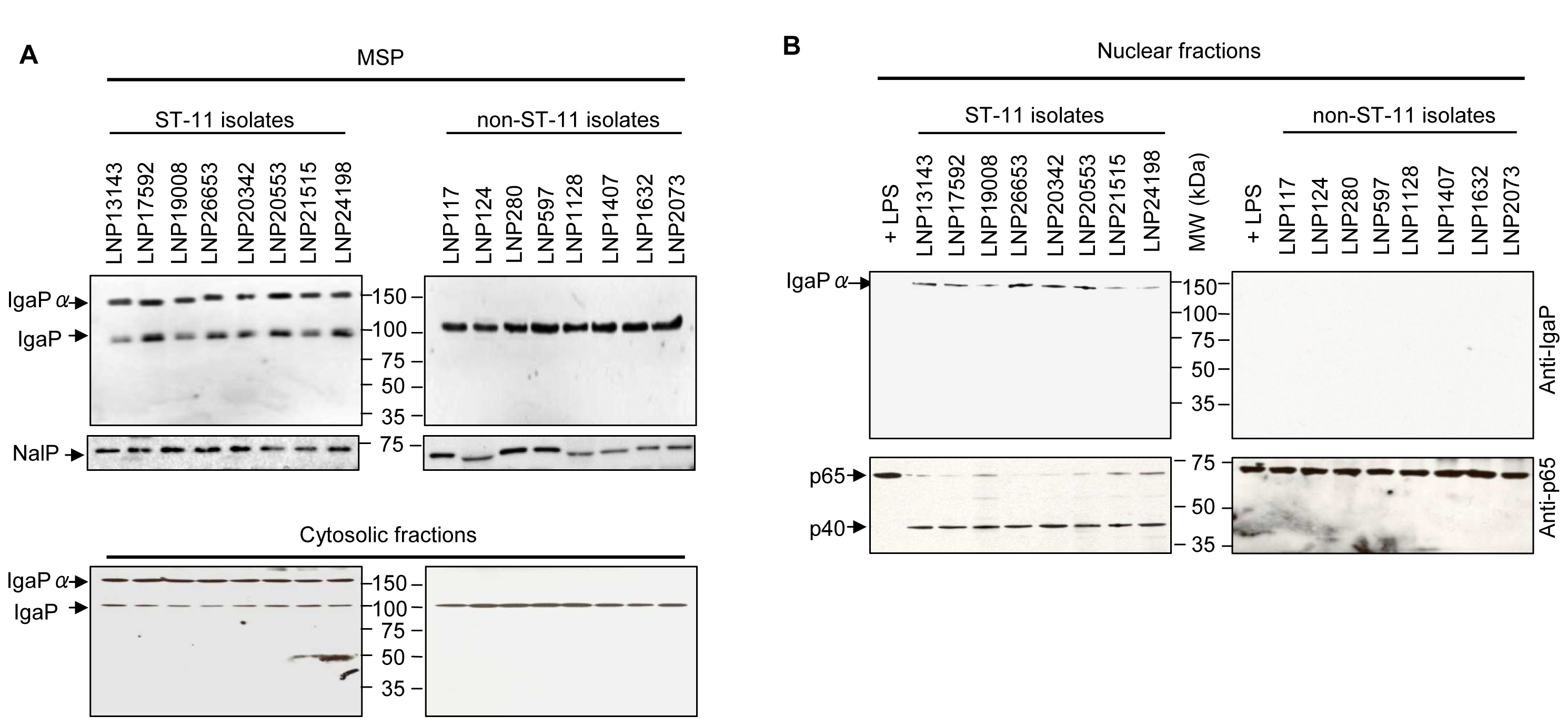

Supplement: S3 Fig — (A) Hec-1-B cells were infected with a subset of ST-11 or non-ST-11 isolates. After 12 h cytosolic fractions were prepared. In parallel, MSP of the indicated isolates were prepared as indicated in Materials and Methods. Samples were resolved by SDS-PAGE and then examined by immunoblot using the polyclonal serum to IgaP or NalP. Positions of both IgA protease secreted forms are indicated by arrows. The molecular weight is presented. (B) The nuclear fractions of infected cells from (A) were prepared, resolved by SDS-PAGE and then immunoblotted with anti-p65 mAb (N-terminal specific) or the polyclonal serum to IgaP. (TIF) [file ppat.1005078.s005.tif]

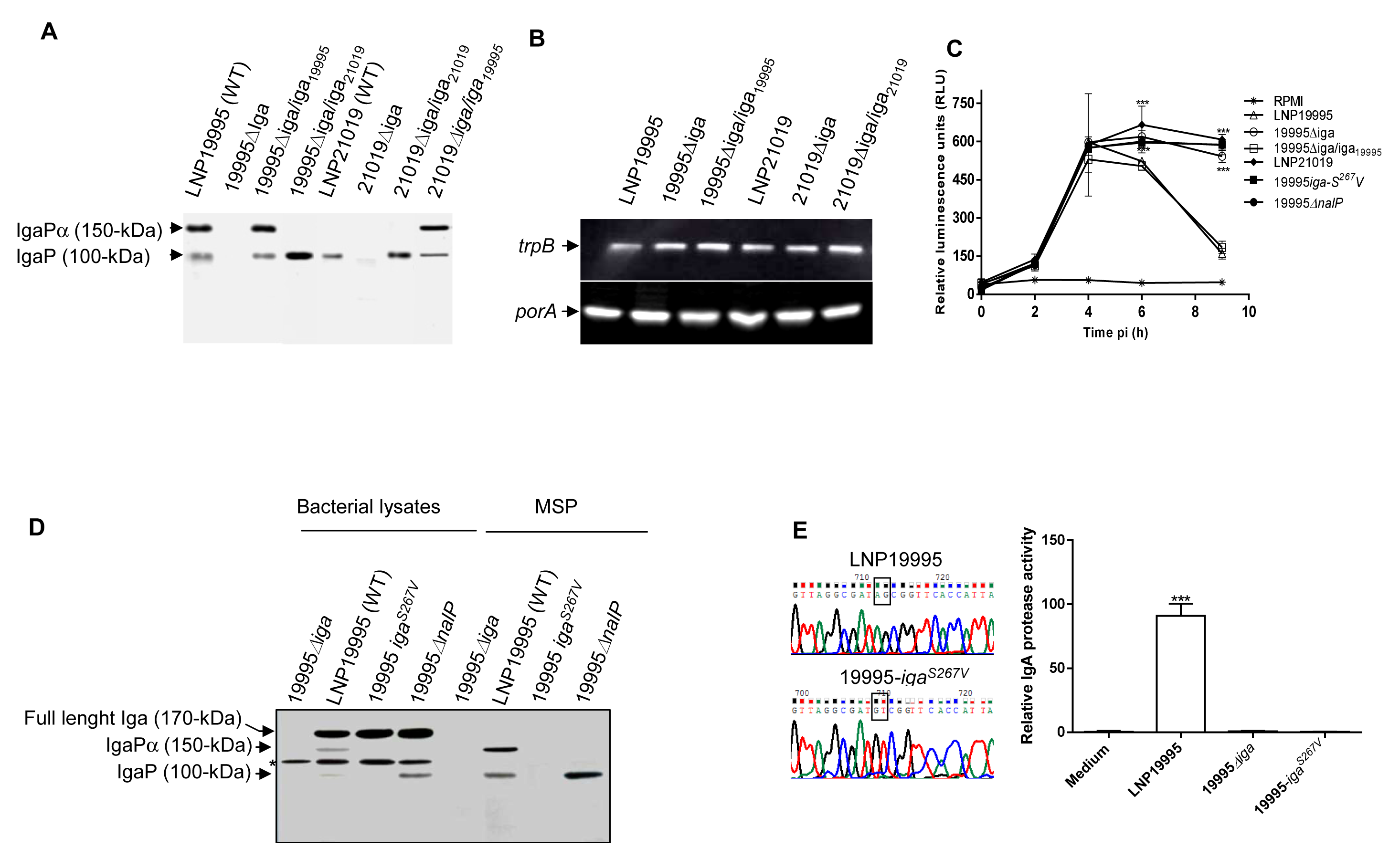

Supplement: S4 Fig — (A) MSPs were prepared from the indicated strains and resolved in SDS-PAGE. After transfer, the membrane was probed against anti-IgaP specific serum. Both IgA protease secreted forms are indicated with arrows. (B) Insertion of spectinomycin resistance cassette in iga has no polar effect on expression of trpB downstream iga. RT-PCR analysis was performed from total RNA extracted from the indicated strains. Samples were analysed on 2.5% agarose gel and stained with ethidium bromide before visualisation by UV transluminator. Expression of porA was used as an internal control. (C) Alteration of NF-κB transcriptional activity is compromised in epithelial cells infected with iga-knock-out mutant of ST-11 isolates. Hec-1-B cells were co-transfected with p(Igκ)3conaluc and pCMVβ prior to infection. After 48 h, cells were infected with the indicated strains. After each time point, cells were harvested and luciferase activities were determined. Data (mean ± SD) are presented as relative luciferase units (RLU). ***, P < 0.001 for a comparison of cells infected with the wild type ST-11 isolate or isogenic complemented strains and those infected with the iga mutant or LNP21019 carriage isolate. (D) Whole bacterial lysates and secreted proteins were prepared from the indicated strains, then resolved in SDS-PAGE and examined by immunobloting using the serum directed against the IgA protease domain. The full length precursor and the cleavage products are indicated by arrows. The molecular sizes of each form is indicated. * represents a cross-reactive band. (E) Left panel: DNA sequence chromatograms of the wild type LNP19995 (WT) and the 19995iga S267V mutant showing the AG to GT substitution (in square). Right panel: Expression of LNP19995 IgA protease WT and mutants 19995Δiga and 19995iga S267V. Enzyme activity was measured by quantitative ELISA with human IgA1 as a substrate, as described in Materials and Methods. Sterile medium as negative control showed no activity. The error b [file ppat.1005078.s006.tif]

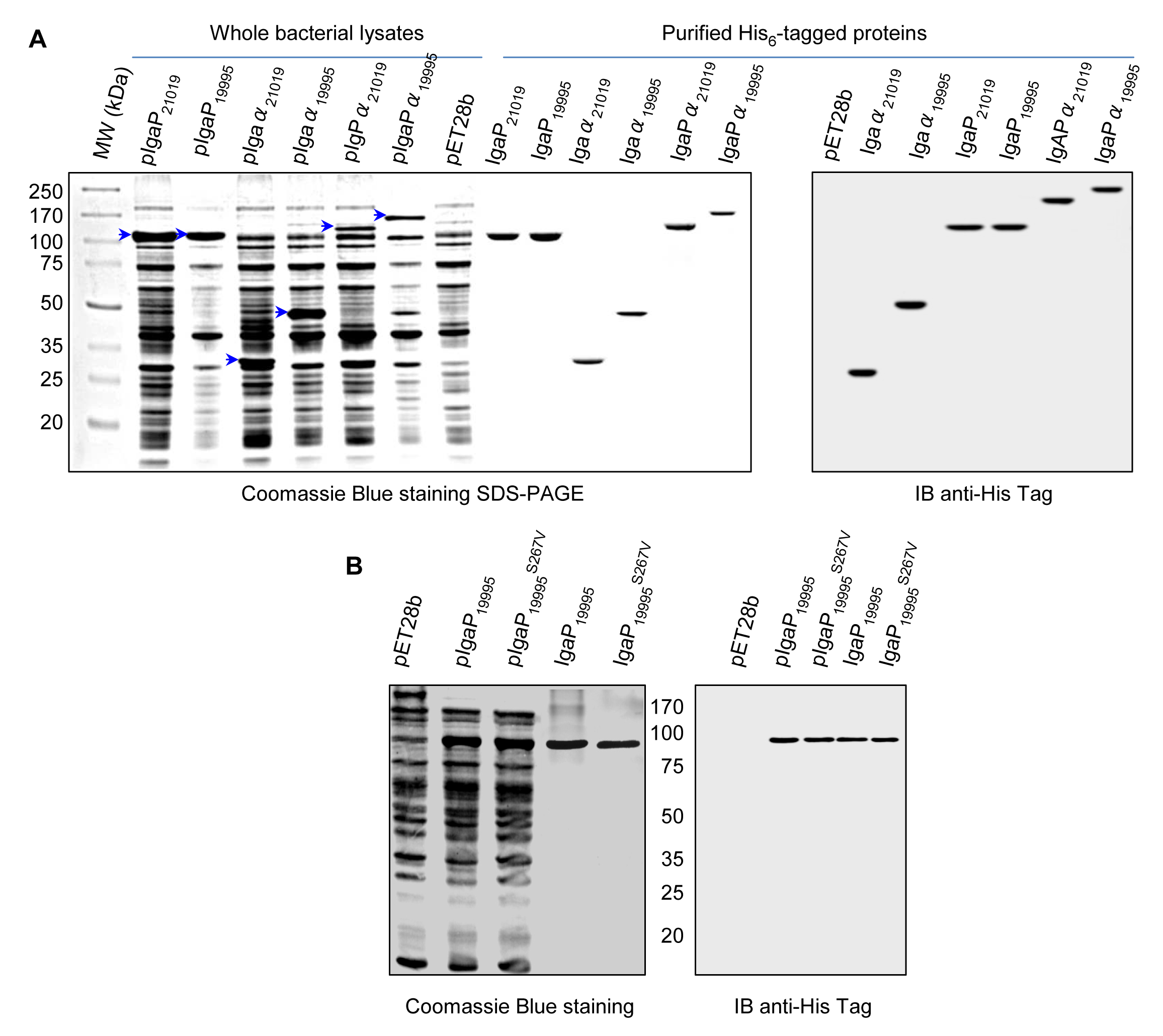

Supplement: S5 Fig — The whole passenger domain IgaPα or each sub-domain IgaP and Igaα (A) and IgaPS267V (B) were subcloned into the vector pET28b from each strain LNP19995 and LNP21019 and were over-expressed and purified from BL21 (DE3) pLyS as C-terminal His6-tagged proteins after induction with 1mM IPTG at 37°C for 2 hours. The total cell lysates and purified proteins were analyzed with SDS-PAGE and blue Coomassie brilliant stain (left panel). Over-expressed proteins are indicated with blue arrowheads. Purified proteins were also confirmed by immunoblotting using anti-His tag specific mAb. The molecular weight (kDa) is shown in the left side. (TIF) [file ppat.1005078.s007.tif]
