# Supplementary material for: Hyperinvasive Meningococci Induce Intra-nuclear Cleavage of the NF-κB Protein p65/RelA by Meningococcal IgA Protease
Source: PLoS Pathog. 2015 Aug 4;11(8):e1005078. doi: 10.1371/journal.ppat.1005078 (PMC4524725; doi:10.1371/journal.ppat.1005078)
Supplement: S6 Fig — The amino acid sequences were deduced from the α-peptide encoding regions of the strains indicated in the left. The alignment was performed with BioEdit program. Gaps, indicated by dashed lines, were introduced by the program. Identical amino acids are in black letters on a yellow background and asterisks in the bottom of alignments. Similar residues are indicated in black letters on grey background. Auto-proteolytic cleavage sequences CS1 and CS2 upstream and downstream α-peptide, respectively, are indicated in black letters on red background. Cleavage sites are indicated by arrowheads. Bipartite NLS sequences are indicated with blue arrows above the sequences. Upstream and downstream sequences of each NLS cluster are indicated in white letters on blue background. The amino acid positions are indicated according to the top sequence. The start of α peptide, the linker and β core (Igaβ) subdomains are indicated. (PDF) [file ppat.1005078.s008.pdf]

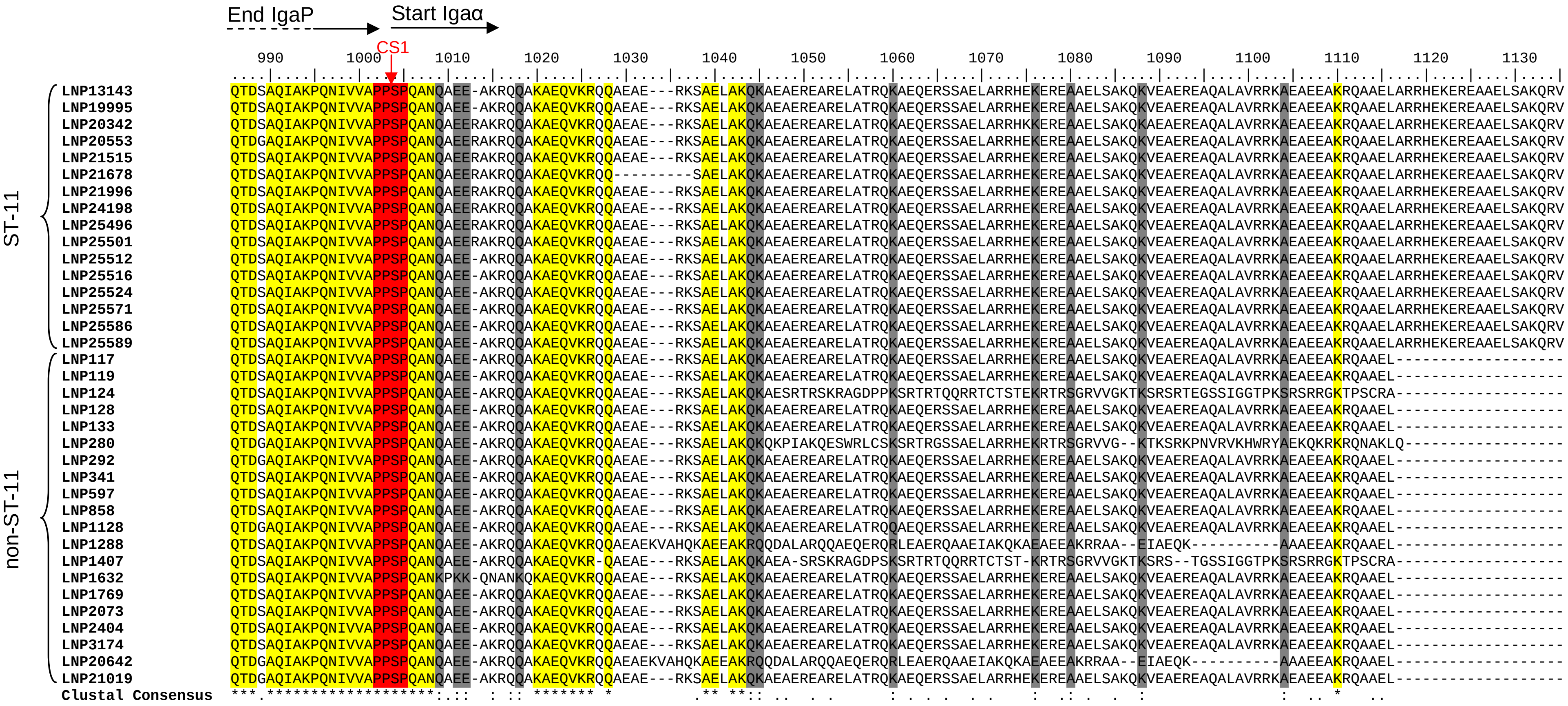

End IgaP
Start Igaα
CS1
ST-11
non-ST-11
LNP13143
LNP19995
LNP20342
LNP20553
LNP21515
LNP21678
LNP21996
LNP24198
LNP25496
LNP25501
LNP25512
LNP25516
LNP25524
LNP25571
LNP25586
LNP25589
LNP117
LNP119
LNP124
LNP128
LNP133
LNP280
LNP292
LNP341
LNP597
LNP858
LNP1128
LNP1288
LNP1407
LNP1632
LNP1769
LNP2073
LNP2404
LNP3174
LNP20642
LNP21019
Clustal Consensus

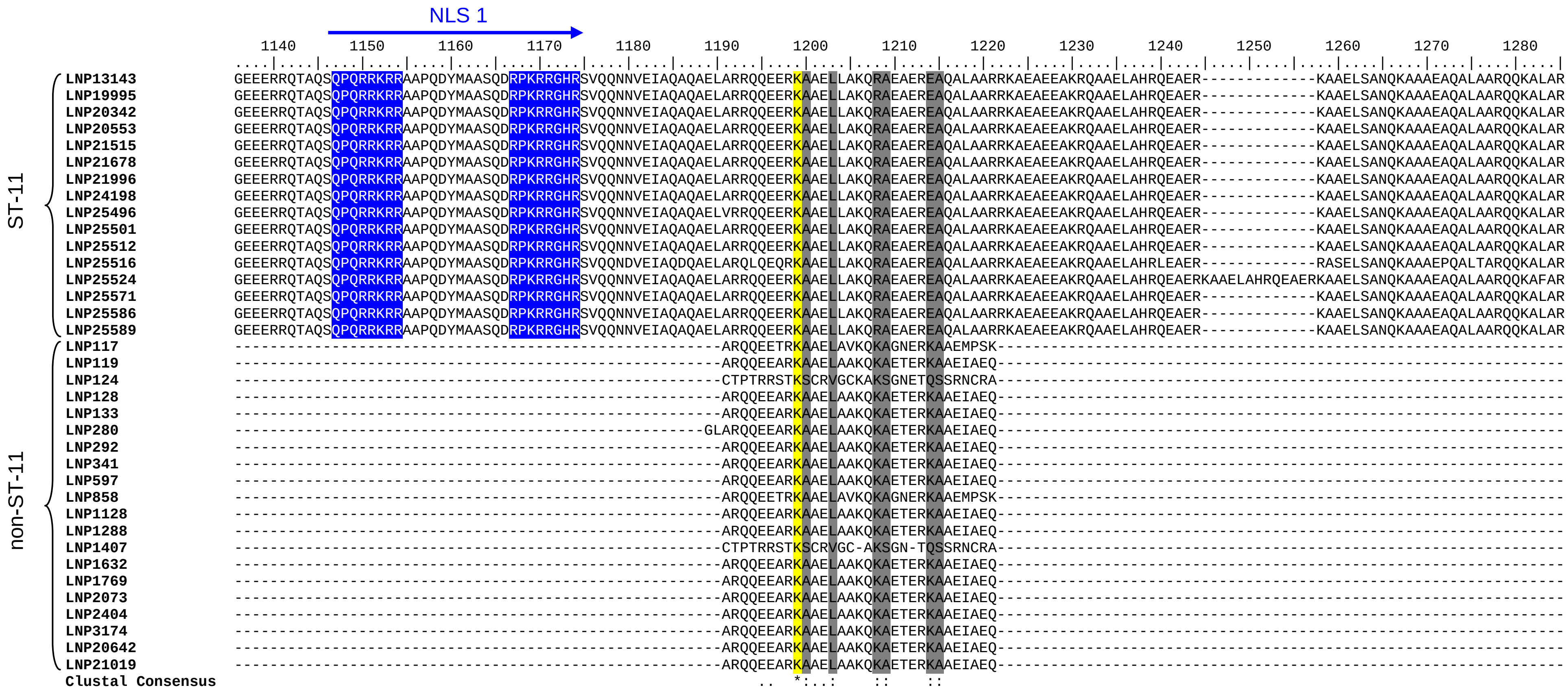
NLS 1
ST-11
non-ST-11
LNP13143
LNP19995
LNP20342
LNP20553
LNP21515
LNP21678
LNP21996
LNP24198
LNP25496
LNP25501
LNP25512
LNP25516
LNP25524
LNP25571
LNP25586
LNP25589
LNP117
LNP119
LNP124
LNP128
LNP133
LNP280
LNP292
LNP341
LNP597
LNP858
LNP1128
LNP1288
LNP1407
LNP1632
LNP1769
LNP2073
LNP2404
LNP3174
LNP20642
LNP21019
Clustal Consensus

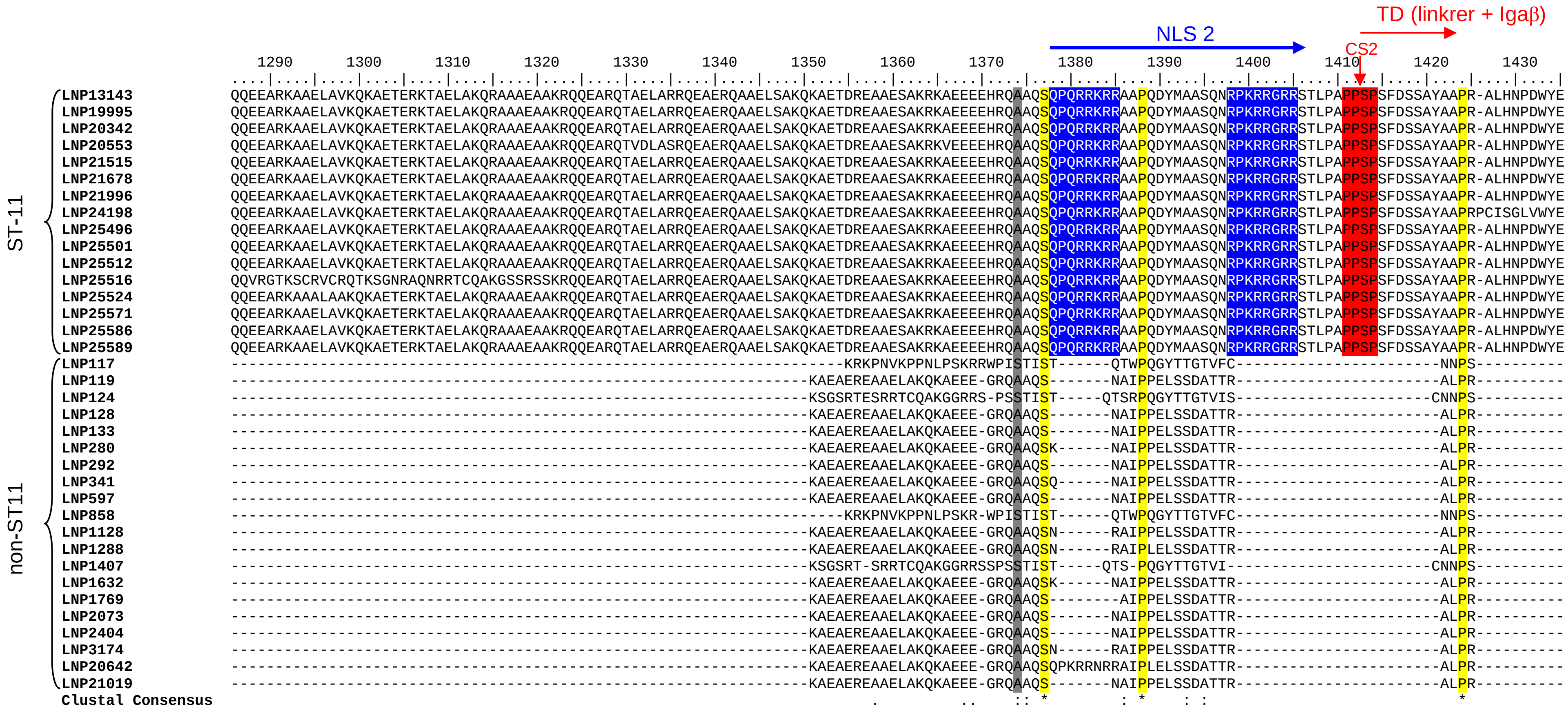
TD (linkrer + Igaβ)
NLS 2
CS2
1290
1300
1310
1320
1330
1340
1350
1360
1370
1380
1390
1400
1410
1420
1430
ST-11
non-ST11
LNP13143
LNP19995
LNP20342
LNP20553
LNP21515
LNP21678
LNP21996
LNP24198
LNP25496
LNP25501
LNP25512
LNP25516
LNP25524
LNP25571
LNP25586
LNP25589
LNP117
LNP119
LNP124
LNP128
LNP133
LNP280
LNP292
LNP341
LNP597
LNP858
LNP1128
LNP1288
LNP1407
LNP1632
LNP1769
LNP2073
LNP2404
LNP3174
LNP20642
LNP21019
Clustal Consensus

```
                      1440      1450      1460      1470      1480      1490      1500      1510      1520      1530      1540      1550      1560      1570      1580
                    ....|....|....|....|....|....|....|....|....|....|....|....|....|....|....|....|....|....|....|....|....|....|....|....|....|....|....|....|....|....|
ST-11
LNP13143            NDYEEIPLDALEDENVSESVDTSDKQPQDNTELHEKYENDYEEIPLDALEDEDVSESVDTSDKQPQDNTELHEKVETVSLQPRAAQPR--------AQAAAQPQA--------QAVAQADAVSTNTNSALSDAMASTQSILLDTGASLTR
LNP19995            NDYEEIPLDALEDENVSESVDTSDKQPQDNTELHEKYENDYEEIPLDALEDEDVSESVDTSDKQPQDNTELHEKVETVSLQPRAAQPR--------AQAAAQPQA--------QAVAQADAVSTNTNSALSDAMASTQSILLDTGASLTR
LNP20342            NDYEEIPLDALEDENVSESVDTSDKQPQDNTELHEKYENDYEEIPLDALEDEDVSESVDTSDKQPQDNTELHEKVETVSLQPRAAQPR--------AQAAAQPQA--------QAVAQADAVSTNTNSALSDAMASTQSILLDTGASLTR
LNP20553            NDYEEIPLDALEDENVSESVDTSDKQPQDNTELHEKYENDYEEIPLDALEDEDVSESVDTSDKQPQDNTELHEKVETVSLQPRAAQPRAQAAAQPQAQAAAQPQA--------QAVAQADAVSTNTNSALSDAMASTQSILLDTGASLTR
LNP21515            NDYEEIPLDALEDENVSESVDTSDKQPQDNTELHEKYENDYEEIPLDALEDEDVSESVDTSDKQPQDNTELHEKVETVSLQPRAAQPRAQAAAQPQAQAAAQPQA--------QAVAQADAVSTNTNSALSDAMASTQSILLDTGASLTR
LNP21678            NDYEEIPLDALEDENVSESVDTSDKQPQDNTELHEKYENDYEEIPLDALEDEDVSESVDTSDKQPQDNTELHEKVETVSLQPRAAQPRAQAAAQPQAQAAAQPQA--------QAVAQADAVSTNTNSALSDAMASTQSILLDTGASLTR
LNP21996            NDYEEIPLDALEDENVSESVDTSDKQPQDNTELHEKYENDYEEIPLDALEDEDVSESVDTSDKQPQDNTELHEKVETVSLQPRAAQPR--------AQAAAQPQ------------AQADAVSTNTNSALSDAMASTQSILLDTGASLTR
LNP24198            NDYEEIPLDALEDENVSESVDTSDKQPQDNTELHEKYENDYEEIPLDALEDEDVSESVDTSDKQPQDNTELHEKVETVSLQPRAAQPRAQAAAQPQAQAAAQPQA--------QAVAQADAVSTNTNSALSDAMASTQSILLDTGASLTR
LNP25496            NDYEEIPLDALEDENVSESVDTSDKQPQDNTELHEKYENDYEEIPLDALEDEDVSESVDTSDKQPQDNTELHEKVETVSLQPRAAQPR--------AQAAAQPQ------------AQADAVSTNTNSALSDAMASTQSILLDTGASLTR
LNP25501            NDYEEIPLDALEDENVSESVDTSDKQPQDNTELHEKYENDYEEIPLDALEDEDVSESVDTSDKQPQDNTELHEKVETVSLQPRAAQPR--------AQAAAQPQA--------QAVAQADAVSTNTNSALSDAMASTQSILLDTGASLTR
LNP25512            NDYEEIPLDALEDENVSESVDTSDKQPQDNTELHEKYENDYEEIPLDALEDEDVSESVDTSDKQPQDNTELHEKVETVSLQPRAAQPR--------AQAAAQPQA--------QAVAQADAVSTNTNSALSDAMASTQSILLDTGASLTR
LNP25516            NDYEEIPLDALEDENVSESVDTSDKQPQDNTELHEKYENDYEEIPLDALEDEDVSESVDTSDKQPQDNTELHEKVETVSLQPRAAQPR--------AQAAAQPQA--------QAVAQADAVSTNTNSALSDAMASTQSILLDTGASLTR
LNP25524            NDYEEIPLDALEDENVSESVDTSDKQPQDNTELHEKYENDYEEIPLDALEDEDVSESVDTSDKQPQDNTELHEKVETVSLQPRAAQPR--------AQAAAQPQA--------QAVAQADAVSTNTNSALSDAMASTQSILLDTGASLTR
LNP25571            NDYEEIPLDALEDENVSESVDTSDKQPQDNTELHEKYENDYEEIPLDALEDEDVSESVDTSDKQPQDNTELHEKVETVSLQPRAAQPR--------AQAAAQPQ------------AQADAVSTNTNSALSDAMASTQSILLDTGASLTR
LNP25586            NDYEEIPLDALEDENVSESVDTSDKQPQDNTELHEKYENDYEEIPLDALEDEDVSESVDTSDKQPQDNTELHEKVETVSLQPRAAQPR--------AQAAAQPQ------------AQADAVSTNTNSALSDAMASTQSILLDTGASLTR
LNP25589            NDYEEIPLDALEDENVSESVDTSDKQPQDNTELHEKYENDYEEIPLDALEDEDVSESVDTSDKQPQDNTELHEKVETVSLQPRAAQPRAQAAAQPQAQAAAQPQA--------QAVAQADAVSTNTNSALSDAMASTQSILLDTGASLTR
non-ST-11
LNP117              -----FADSEKFK------------------------SCQRLRNPLGCVGR---GRGIRIGHIRQTASRQYGTSKSDSELQPRAAQPR--------AQAAAQPQAQAAAQPQAQAVAQADAVSTNTNSALSDAMASTQSILLDTGASLTR
LNP119              -----IARNSNPD------------------------ASDYEEIPLDALEDEDVSESVDTSDKQPQDNTELHEKVETVSLQPRAAQPR--------AQAAAQPQAQAAAQPQAQAVAQADAVSTNTNSALSDAMASTQSILLDTGASLTR
LNP124              -----FALDSEKFKSN-------------------CQRLN-RNIPLDALEDEDVSESVDTSDKQPQDNTELHEKVETVSLQPRAAQPR--------AQAAAQPQAQAAAQPQAQAVAQADAVSTNTNSALSDAMASTQSILLDTGASLTR
LNP128              -----IARNSNPD------------------------ASDYEEIPLDALEDEDVSESVDTSDKQPQDNTELHEKVETVSLQPRAAQPR--------AQAAAQPQAQAAAQPQAQAVAQADAVSTNTNSALSDAMASTQSILLDTGASLTR
LNP133              -----IARNSNPD------------------------ASDYEEIPLDALEDEDVSESVDTSDKQPQDNTELHEKVETVSLQPRAAQPR--------AQAAAQPQAQAAAQPQAQAVAQADAVSTNTNSALSDAMASTQSILLDTGASLTR
LNP280              -----IARNSNPD------------------------ASDYEEIPLDALEDEDVSESVDTSDKQPQDNTELHEKVETVSLQPRAAQPR--------AQAAAQP------------QAQADAVSTNTNSALSDAMASTQSILLDTGASLTR
LNP292              -----IARNSNPD------------------------ASDYEEIPLDALEDEDVSESVDTSDKQPQDNTELHEKVETVSLQPRAAQPR--------AQAAAQP------------QAQADAVSTNTNSALSDAMASTQSILLDTGASLTR
LNP341              -----IARNSNPD------------------------ASDYEEIPLDALEDEDVSESVDTSDKQPQDNTELHEKVETVSLQPRAAQPR--------AQAAAQP--------QAQAVAQADAVSTNTNSALSDAMASTQSILLDTGASLTR
LNP597              -----IARNSNPD------------------------ASDYEEIPLDALEDEDVSESVDTSDKQPQDNTELHEKVETVSLQPRAAQPR--------AQAAAQP------------QAQADAVSTNTNSALSDAMASTQSILLDTGASLTR
LNP858              -----FADSEKFK------------------------SCQRLRNPLGCVGR---GRGIRIGHIRQTASRQYGTSKSDSELQPRAAQPR--------AQAAAQP--------QAQAVAQADAVSTNTNSALSDAMASTQSILLDTGASLTR
LNP1128             -----IARNSNPD------------------------ASDYEEIPLDALEDEDVSESVDTSDKQPQDNTELHEKVETVSLQPRAAQPR--------AQAAAQP--------QAQAVAQADAVSTNTNSALSDAMASTQSILLDTGASLTR
LNP1288             -----IARNSNPD------------------------ASDYEEIPLDALEDEDVSESVDTSDKQPQDNTELHEKVETVSLQPRAAQPR--------AQAAAQP------------QAQADAVSTNTNSALSDAMASTQSILLDTGASLTR
LNP1407             -----FA-DSEKFKS--------------------CQRLDYEEIPLDALEDEDVSESVDTSDKQPQDNTELHEKVETVSLQPRAAQPR--------AQAAAQPQAQ------------ADAVSTNTNSALSDAMASTQSILLDTGASLTR
LNP1632             -----IARNSNPD------------------------ASDYEEIPLDALEDEDVSESVDTSDKQPQDNTELHEKVETVSLQPRAAQPR--------AQAAAQPQAQAAAQPQAQAVAQADAVSTNTNSALSDAMASTQSILLDTGASLTR
LNP1769             -----IARNSNPD------------------------ASDYEEIPLDALEDEDVSESVDTSDKQPQDNTELHEKVETVSLQPRAAQPR--------AQAAAQPQAQAAAQPQAQAVAQADAVSTNTNSALSDAMASTQSILLDTGASLTR
LNP2073             -----IARNSNPD------------------------ASDYEEIPLDALEDEDVSESVDTSDKQPQDNTELHEKVETVSLQPRAAQPR--------AQAAAQP------------QAQADAVSTNTNSALSDAMASTQSILLDTGASLTR
LNP2404             -----IARNSNPD------------------------ASDYEEIPLDALEDEDVSESVDTSDKQPQDNTELHEKVETVSLQPRAAQPR--------AQAAAQP------------QAQADAVSTNTNSALSDAMASTQSILLDTGASLTR
LNP3174             -----IARNSNPD------------------------ASDYEEIPLDALEDEDVSESVDTSDKQPQDNTELHEKVETVSLQPRAAQPR--------AQAAAQP--------QA----QADAVSTNTNSALSDAMASTQSILLDTGASLTR
LNP20642            -----IARNSNPD------------------------ASDYEEIPLDALEDEDVSESVDTSDKQPQDNTELHEKVETVSLQPRAAQPR--------AQAAAQP------------QAQADAVSTNTNSALSDAMASTQSILLDTGASLTR
LNP21019            -----IARNSNPD------------------------ASDYEEIPLDALEDEDVSESVDTSDKQPQDNTELHEKVETVSLQPRAAQPR--------AQAAAQP--------QAQAVAQADAVSTNTNSALSDAMASTQSILLDTGASLTR
Clustal Consensus        :. .                              :  . **..:     ...:  .. :   . :   . .  .*********        *******               ********************************
```

```
                      1590      1600      1610      1620      1630      1640      1650      1660      1670
                  ....|....|....|....|....|....|....|....|....|....|....|....|....|....|....|....|....|....|..
ST-11
LNP13143          HIAQKSRADAEKNSVWMSNIGYGRDYASAQYRRFSSKRTQTQIGIDRSLSENMQIGGVLTYSDSQHTFDQASGKNTFVQANLYGKYYLNDAW
LNP19995          HIAQKSRADAEKNSVWMSNIGYGRDYASAQYRRFSSKRTQTQIGIDRSLSENMQIGGVLTYSDSQHTFDQASGKNTFVQANLYGKYYLNDAW
LNP20342          HIAQKSRADAEKNSVWMSNIGYGRDYASAQYRRFSSKRTQTQIGIDRSLSENMQIGGVLTYSDSQHTFDQASGKNTFVQANLYGKYYLNDAW
LNP20553          HIAQKSRADAEKNSVWMSNIGYGRDYASAQYRRFSSKRTQTQIGIDRSLSENMQIGGVLTYSDSQHTFDQASGKNTFVQANLYGKYYLNDAW
LNP21515          HIAQKSRADAEKNSVWMSNIGYGRDYASAQYRRFSSKRTQTQIGIDRSLSENMQIGGVLTYSDSQHTFDQASGKNTFVQANLYGKYYLNDAW
LNP21678          HIAQKSRADAEKNSVWMSNIGYGRDYASAQYRRFSSKRTQTQIGIDRSLSENMQIGGVLTYSDSQHTFDQASGKNTFVQANLYGKYYLNDAW
LNP21996          HIAQKSRADAEKNSVWMSNIGYGRDYASAQYRRFSSKRTQTQIGIDRSLSENMQIGGVLTYSDSQHTFDQASGKNTFVQANLYGKYYLNDAW
LNP24198          HIAQKSRADAEKNSVWMSNIGYGRDYASAQYRRFSSKRTQTQIGIDRSLSENMQIGGVLTYSDSQHTFDQASGKNTFVQANLYGKYYLNDAW
LNP25496          HIAQKSRADAEKNSVWMSNIGYGRDYASAQYRRFSSKRTQTQIGIDRSLSENMQIGGVLTYSDSQHTFDQASGKNTFVQANLYGKYYLNDAW
LNP25501          HIAQKSRADAEKNSVWMSNIGYGRDYASAQYRRFSSKRTQTQIGIDRSLSENMQIGGVLTYSDSQHTFDQASGKNTFVQANLYGKYYLNDAW
LNP25512          HIAQKSRADAEKNSVWMSNIGYGRDYASAQYRRFSSKRTQTQIGIDRSLSENMQIGGVLTYSDSQHTFDQASGKNTFVQANLYGKYYLNDAW
LNP25516          HIAQKSRADAEKNSVWMSNIGYGRDYASAQYRRFSSKRTQTQIGIDRSLSENMQIGGVLTYSDSQHTFDQASGKNTFVQANLYGKYYLNDAW
LNP25524          HIAQKSRADAEKNSVWMSNIGYGRDYASAQYRRFSSKRTQTQIGIDRSLSENMQIGGVLTYSDSQHTFDQASGKNTFVQANLYGKYYLNDAW
LNP25571          HIAQKSRADAEKNSVWMSNIGYGRDYASAQYRRFSSKRTQTQIGIDRSLSENMQIGGVLTYSDSQHTFDQASGKNTFVQANLYGKYYLNDAW
LNP25586          HIAQKSRADAEKNSVWMSNIGYGRDYASAQYRRFSSKRTQTQIGIDRSLSENMQIGGVLTYSDSQHTFDQASGKNTFVQANLYGKYYLNDAW
LNP25589          HIAQKSRADAEKNSVWMSNIGYGRDYASAQYRRFSSKRTQTQIGIDRSLSENMQIGGVLTYSDSQHTFDQASGKNTFVQANLYGKYYLNDAW
non-ST-11
LNP117            HIAQKSRADAEKNSVWMSNIGYGRDYASAQYRRFSSKRTQTQIGIDRSLSENMQIGGVLTYSDSQHTFDQASGKNTFVQANLYGKYYLNDAW
LNP119            HIAQKSRADAEKNSVWMSNIGYGRDYASAQYRRFSSKRTQTQIGIDRSLSENMQIGGVLTYSDSQHTFDQASGKNTFVQANLYGKYYLNDAW
LNP124            HIAQKSRADAEKNSVWMSNIGYGRDYASAQYRRFSSKRTQTQIGIDRSLSENMQIGGVLTYSDSQHTFDQASGKNTFVQANLYGKYYLNDAW
LNP128            HIAQKSRADAEKNSVWMSNIGYGRDYASAQYRRFSSKRTQTQIGIDRSLSENMQIGGVLTYSDSQHTFDQASGKNTFVQANLYGKYYLNDAW
LNP133            HIAQKSRADAEKNSVWMSNIGYGRDYASAQYRRFSSKRTQTQIGIDRSLSENMQIGGVLTYSDSQHTFDQASGKNTFVQANLYGKYYLNDAW
LNP280            HIAQKSRADAEKNSVWMSNIGYGRDYASAQYRRFSSKRTQTQIGIDRSLSENMQIGGVLTYSDSQHTFDQASGKNTFVQANLYGKYYLNDAW
LNP292            HIAQKSRADAEKNSVWMSNIGYGRDYASAQYRRFSSKRTQTQIGIDRSLSENMQIGGVLTYSDSQHTFDQASGKNTFVQANLYGKYYLNDAW
LNP341            HIAQKSRADAEKNSVWMSNIGYGRDYASAQYRRFSSKRTQTQIGIDRSLSENMQIGGVLTYSDSQHTFDQASGKNTFVQANLYGKYYLNDAW
LNP597            HIAQKSRADAEKNSVWMSNIGYGRDYASAQYRRFSSKRTQTQIGIDRSLSENMQIGGVLTYSDSQHTFDQASGKNTFVQANLYGKYYLNDAW
LNP858            HIAQKSRADAEKNSVWMSNIGYGRDYASAQYRRFSSKRTQTQIGIDRSLSENMQIGGVLTYSDSQHTFDQASGKNTFVQANLYGKYYLNDAW
LNP1128           HIAQKSRADAEKNSVWMSNIGYGRDYASAQYRRFSSKRTQTQIGIDRSLSENMQIGGVLTYSDSQHTFDQASGKNTFVQANLYGKYYLNDAW
LNP1288           HIAQKSRADAEKNSVWMSNIGYGRDYASAQYRRFSSKRTQTQIGIDRSLSENMQIGGVLTYSDSQHTFDQASGKNTFVQANLYGKYYLNDAW
LNP1407           HIAQKSRADAEKNSVWMSNIGYGRDYASAQYRRFSSKRTQTQIGIDRSLSENMQIGGVLTYSDSQHTFDQASGKNTFVQANLYGKYYLNDAW
LNP1632           HIAQKSRADAEKNSVWMSNIGYGRDYASAQYRRFSSKRTQTQIGIDRSLSENMQIGGVLTYSDSQHTFDQASGKNTFVQANLYGKYYLNDAW
LNP1769           HIAQKSRADAEKNSVWMSNIGYGRDYASAQYRRFSSKRTQTQIGIDRSLSENMQIGGVLTYSDSQHTFDQASGKNTFVQANLYGKYYLNDAW
LNP2073           HIAQKSRADAEKNSVWMSNIGYGRDYASAQYRRFSSKRTQTQIGIDRSLSENMQIGGVLTYSDSQHTFDQASGKNTFVQANLYGKYYLNDAW
LNP2404           HIAQKSRADAEKNSVWMSNIGYGRDYASAQYRRFSSKRTQTQIGIDRSLSENMQIGGVLTYSDSQHTFDQASGKNTFVQANLYGKYYLNDAW
LNP3174           HIAQKSRADAEKNSVWMSNIGYGRDYASAQYRRFSSKRTQTQIGIDRSLSENMQIGGVLTYSDSQHTFDQASGKNTFVQANLYGKYYLNDAW
LNP20642          HIAQKSRADAEKNSVWMSNIGYGRDYASAQYRRFSSKRTQTQIGIDRSLSENMQIGGVLTYSDSQHTFDQASGKNTFVQANLYGKYYLNDAW
LNP21019          HIAQKSRADAEKNSVWMSNIGYGRDYASAQYRRFSSKRTQTQIGIDRSLSENMQIGGVLTYSDSQHTFDQASGKNTFVQANLYGKYYLNDAW
Clustal Consensus ********************************************************************************************
```
